# circCYP24A1 promotes Docetaxel resistance in prostate Cancer by Upregulating ALDH1A3

**DOI:** 10.1186/s40364-022-00393-1

**Published:** 2022-07-13

**Authors:** Haoli Yin, Haixiang Qin, Lei Yang, Mengxia Chen, Yang Yang, Wenlong Zhang, Jiange Hao, Qun Lu, Jingyan Shi, Junlong Zhuang, Xuefeng Qiu, Hongqian Guo

**Affiliations:** 1 Department of Urology, Affiliated Drum Tower Hospital, Medical School of Nanjing University, Nanjing, 210008 China; 2 Institute of Urology, Nanjing University, Nanjing, China; 3 Immunology and Reproduction Biology Laboratory & State Key Laboratory of Analytical Chemistry for Life Science, Medical School, Nanjing University, Nanjing, 210093 Jiangsu China; 4 Jiangsu Key Laboratory of Molecular Medicine, Nanjing University, Nanjing, 210093 Jiangsu China; 5 Department of Urology, The Second Affiliated Hospital of Dalian Medical University, Dalian, 116000 China

**Keywords:** Prostate cancer, circCYP24A1, miR-1301-3p, ALDH1A3, Docetaxel resistance

## Abstract

**Background:**

Docetaxel (DTX) is the most widely prescribed first-line chemotherapy for advanced prostate cancer (PCa). Unfortunately, DTX resistance invariably emerges, leading to worse prognosis of PCa. Growing evidence has shown that circRNAs had complex spatiotemporal specificity during the tumor development and oncogenesis. This study was designed to investigate the biological functions and possible molecular mechanisms of circRNAs in DTX resistance of PCa.

**Methods:**

circRNAs in established DTX-resistant DU145 cell line were identified by RNA sequencing. Biological function of circCYP24A1 was verified in vitro and in vivo. The potential role of circCYP24A1 in the development of DTX-resistant PCa was investigated via dual-luciferase reporter assays, RIP assays and RNA pull-down assays. Univariate and multivariate logistic regression analyses was used to predict DTX-chemotherapy response based on patients’ clinical and biological information.

**Results:**

CircCYP24A1 was identified to be upregulated in DTX-resistant DU145 cells. Upregulated circCYP24A1 was found to suppress the DTX chemosensitivity in vitro and in vivo. Furthermore, we found that circCYP24A1 promoted DTX resistance in PCa via regulating ALDH1A3 expression by sponging miR-1301-3p and activating PI3K/AKT/mTOR signaling pathway. Statistical analyses elucidated that circCYP24A1 was an independent risk factor to predict DTX response (OR = 0.165; 95% CI: 0.038–0.723; *P* = 0.017).

**Conclusions:**

This study demonstrated that circCYP24A played an essential role in DTX resistance in PCa, suggesting that circCYP24A1 could be a promising biomarker to predict DTX response and a potential therapeutic target in PCa patients resistant to DTX chemotherapy.

**Supplementary Information:**

The online version contains supplementary material available at 10.1186/s40364-022-00393-1.

## Background

Prostate cancer (PCa) has the greatest morbidity in aging men [1]. Notwithstanding benefitted from early diagnosis and locoregional therapy, 15% patients present with high-risk, local disease will progress to a metastatic stage and remain a pressing need for systemic therapy [2]. Attributed to the development of therapies for advanced PCa, docetaxel (DTX) is recommended as the first-line therapy applied in the administration of metastatic hormone-sensitive PCa (mHSPC) [3], as well as metastatic castration-resistant PCa (mCRPC) in combination with continuous androgen deprivation therapy [4–6].

Unfortunately, DTX resistance invariably emerges, leading to worse prognosis of PCa. In clinical practice, tumor volume (High vs. Low volume) on the conventional images was used to predict the benefits of DTX-based therapy for patients with mHSPC. However, this was just based on the subgroup analyses from two prospective trails [5, 7], and the definition of high or low volume of mHSPC remains controversial [8, 9]. Recently, genetic biomarkers have been shown to be promising in selecting mCRPC patients who might get benefits from PARP inhibitor and AKT inhibitor [10–12]. However, no genetic biomarkers have not been proven to be useful in predicting DTX response. Thus, development of biomarkers which is able to predict the benefits from DTX-based chemotherapy and novel strategies targeting signaling pathways involved in DTX resistance are urgently warranted in PCa patients.

Accumulating evidence descripts that the non-coding portion of the transcriptome is crucial for homoeostasis, metabolism, and cell fate [13, 14]. Among them, the expression of circular RNAs (circRNAs) shows complex spatiotemporal specificity during the development and oncogenesis of eukaryotes [15–18]. The closed-loop structure confers much more stability to circRNAs compared with linear RNAs [19]. Increasing number of researches have indicated the role of circRNAs in a variety of neoplastic processes, such as tumor development, tumor recurrence and chemotherapy resistance [20–22]. CircRNAs affect the oncogenesis by serving as miRNA sponges, which could mediate the progression of cancer via uregulating the downstream mRNA. For instance, the first characterized circRNA, ciRS-7, has been well verified to sponge miR-7 and inhibit the expression of miR-7 in diverse cancers [23].

In this work, we found that circCYP241, known as has_circ_0060927, was vital for generation and maintenance of DTX-resistant PCa. Our data illustrated that circCYP241 was highly expressed in DTX-resistant PCa cells. Down-regulation of circCYP241 notably increased DTX-induced cell death. Molecular mechanistic studies determined that circCYP241 bound with miR-1301-3p in the cytosol, and regulated ALDH1A3 expression through post-transcriptional manner. Clinical analysis elucidated that circCYP24A1 could be used to predict DTX response.

## Methods

### Cell culture and resistant cell line development

HEK293T, DU145 and 22Rv1 cell lines were purchased from the Cell Bank of Type Culture Collection, Chinese Academy of Science (Shanghai, China). All cell lines were determined for mycoplasma using MycAway™ Plus-Color One-Step Mycoplasma Detection Kit (YEASEN, Shanghai, China). The cells were incubated in RPMI-1640 medium (WISENT, Montreal, Canada) supplemented with 10% FBS (Gibco, Grand Island, NY) and 1% penicillin-streptomycin (WISENT), and maintained at 37 °C in a humidified 5% CO_2_ atmosphere.

The DTX-resistant DU145 (DU145-DR) sub-lines were developed by stepwise increased concentrations of DTX, essentially as described in the following. Parental DU145 cells were treated with DTX (MCE, Monmouth Junction, NJ) suspended in DMSO at an initial concentration of 2 nM for 2 days. The surviving cells were collected and seeded into new dish after one-cycle treatment. As DU145 cells shown resistance, the concentration of DTX was subsequently increased to 100 nM. Before increasing drug concentration or any experiment, cells were allowed to completely recover. Decreased death and increased proliferation indicated the adaptation of cells to DTX. The DTX-resistant sub-cell lines were continuously maintained in 10 nM DTX, and media containing DTX was freshened if necessary. The parental cells were cultured and passaged along with the construction of DU145-DR. Batches of cells were cryopreserved, and assays were conducted on same passages [24].

### Tissue specimens and patients’ chemotherapy response assessment

PCa tissue specimens, including resistant and sensitive to DTX, were obtained from 70 patients receiving 6-cycles neoadjuvant treatment (goserelin plus DTX and prednisone) in an institutional board-approved research protocol (clinicaltrials.gov identification ID: NCT04869371) at Nanjing Drum Tower Hospital from Jan 2019 to Dec 2020. All patients received radical prostatectomy after neoadjuvant therapy. All patients provided written informed consent before treatment. The corresponding clinical, radiological and pathological characteristics were collected as well.

The whole-mount pathologic results were recognized as golden standard for assessing neoadjuvant treatment response. Specifically, the largest cross-section bidimensional in postoperative pathologic results shorter than 5 mm were defined as pathologic complete response (pCR) or minimal residual disease (MDR) [25]. In this study, DTX-sensitive was defined as patients with pCR or MDR and the other were regarded as DTX-resistant. This study was undertaken with the understanding and written consent of each subject, with approval of the Ethics Committee of Drum Tower Hospital, Medical School of Nanjing University (Nanjing, China), and in compliance with the principles of the 1964 Declaration of Helsinki.

### RNA sequencing

Total RNA was purified from three generations of DU145-DR cells by TRIzol Reagent (Vazyme Biotech Co.,Ltd., Nanjing, China), and the RNA from aging-matched parental cells was set as control. Ribosomal RNA was diminished by RiboMinus Eukaryote kit (Qiagen, Valencia, CA) before establishing the library. Then, the Illumina HiSeq 2000 instrument (Illumina, San Diego, CA) was used to deep sequence the information from the RNA-seq library. The data of RNA-seq were first mapped to the hg38 human reference genome using TopHat2. After dropping out the sequences, which are along to the genome contiguously, the remaining reads were used to characterize circRNAs.

### RNA isolation and quantitative realtime PCR (qRT-PCR) assays

TRIzol reagent (Vazyme) was utilized to purify RNA from cell lines following the manufacturers’ protocol. Cytoplasmic and nuclear RNA was extracted applied a Cytoplasmic & Nuclear RNA Purification Kit (Norgen Biotek, Thorold, ON, Canada) following the product description. PrimeScript® RT Reagent Kit with gDNA eraser (Takara, Kusatsu, Japan) were applied for producing cDNA from total RNA. qRT-PCR assays were conducted using SYBR® Premix ExTaq (TaKaRa), and data was collected by the StepOnePlus™ Real-Time PCR System (Applied Biosystems, CA). Otherwise, the expression of miRNAs was quantified by miRNA Universal SYBR qPCR Master Mix (Vazyme) and miRNA 1st Strand cDNA Synthesis Kit (Vazyme) was applied for the single-stranded cDNA synthesis. U6 snRNA was chosen as a loading control to normalize the expression of miRNAs and ACTB mRNA for mRNAs and circRNAs. All the primers for RNAs are listed in Additional file 8: Table S1.

### RNA stability assays

Total RNA was treated with 3 U/μg RNase R (Epicentre Technologies, Madison, WI, USA) for 15 min at 37 °C, and RNA was purified for cDNA synthesis. Cells were seeded into dishes and added 5 μg/ml actinomycin D (ActD, MCE) to block transcription for the corresponding time period. The level of circCYP24A1 and CYP24A1 mRNA was analyzed through qRT-PCR.

### RNA immunoprecipitation (RIP) assays

The RIP assays were performed with Magna RIP RNA-binding protein immunoprecipitation kit (Bersinbio) following the manufacturer’s protocol. The indicated cells (2X10^7^) were lysed in complete RIP lysis buffer and incubated with human anti-Argonaute2 (AGO2, CST, USA) or non-specific anti-immunoglobulin G (IgG, Millipore) antibody-conjugated beads in rotation for 12 hours at 4 °C. The enriched RNA was obtained from the binding complexes by completely washed, eluted and purified. The enrichments of the target RNAs were detected by qRT-PCR.

### RNA pull-down assays

The biotinylated RNA probes (circCYP24A1 and miR-1301-3p) were purchased from GenePharma Technology (Shanghai, China). RNA pull-down assay was conducted with an RNA Antisense Purification (RAP) Kit (BersinBio, Guangzhou, China) following the product manual. The RNA enrichment levels were analyzed by qRT-PCR and normalized to input group. The probes are listed in Additional file 9: Table S2.

### Dual-luciferase reporter assay

The potential miR-1301-3p binding site of circCYP24A1 and its mutant were synthesized and subcloned into pGLO-miR plasmids respectively, named as pGLO-circCYP24A1-wild-type (WT) and pGLO-circCYP24A1 mutant (MUT). As the same, the 3′-UTR of ALDH1A3 containing the predicted miR-1301-3p-binding sites or mutant sites was amplified and cloned into pGLO-miR, named as pGLO-ALDH1A3-WT and pGLO-ALDH1A3-MUT. HEK293T cells were co-transfected with indicated luciferase plasmids and mimics. Firefly luciferase activity and Renilla luciferase activity were determined after transfected 48 h with a Dual-Luciferase reporter system (Vazyme), and Renilla luciferase activity was set as internal control.

### Protein extraction and western blot

Cells were washed with pre-cooled wash buffer for 3 times and lysed in RIPA buffer (abcam, USA) containing protease inhibitor cocktail (Thermo Fisher Scientific, Waltham, MA) and phosphatase inhibitor cocktail (Thermo Fisher Scientific). Protein content was detected by bicinchoninic acid (BCA) protein assay kit (Vazyme) after centrifugation. The soluble fractions were mixed with 5 × loading buffer and boiled for 5 min. Protein samples were separated by SDS-PAGE and transferred to PVDF membranes (Bio-Rad Laboratories, Hercules, CA, USA). After blocked with 5% (w/v) non-fat milk, the membranes were incubated with appropriately diluted primary antibodies overnight at 4 °C. Horseradish peroxidase (HRP)-conjugated secondary antibodies were probed 1 h at 25 °C. The signals were visualized with ECL reagent (Vazyme), and the intensity values of protein bands were normalized to ACTB. The information about antibodies is showed in Additional file 10: Table S3.

### Flow cytometry

Underwent different condition, cells were collected and stained with propidium iodide (PI) and APC-conjugated Annexin V as the product manual (MutiSciences, AP107-V01). Similarly, cell cycle could be detected based on the reagents from a PI/RNase Staining Kit (BD Biosciences). The proportion of apoptosis cells and cell cycle were then analyzed using flow cytometry (BD Biosciences).

### CCK-8 assay

CCK-8 assay (Vazyme) was applied for assessment of cell survival. Cells (4 × 10^3^) were seeded into 96-well plates and the DTX was added. CCK-8 working solution was added in after treating with DTX for 2 days. After incubating for 90 min at 37 °C, the absorbance at 450 nm was measured using a microplate reader (TECAN Group Ltd., Infinite M200pro, Mannedorf, CH/CHE, Switzerland).

### Immunohistochemistry (IHC)

Clinical PCa specimens and xenografts were used for IHC following the product manual. Paraffin-embedded sections were deparaffinized through a standard procedure. HRP-conjugated species-specific antibodies were incubated in the sections after three times PBST washing. The final signals were detected using DAB Substrate Kit (Servicebio, Wuhan, China) according to the manufacturer’s protocol. For clinical PCa tissues, the staining intensity of ALDH1A3 were scored as 0 (low), 1 (moderate), and 2 (high), and the staining range of tumor was scored as 0 (0–25%), 1 (25–50%), 2 (50–75%), and 3 (75–100%) independently by two well-trained pathologists. The final score was the summation of staining intensity score and staining range score. Low expression was set as the summed scores score ≤ 3 while > 3 as high expression. Images were captured by applying a digital microscope camera (LEICA DM 1000, Nussloch, Germany). The details about antibodies are displayed in Additional file 10: Table S3.

### TUNEL assay

To determine the apoptosis levels, xenografts slices of each mouse were prepared and stained using a TUNEL assay kit (Roche Applied Science, Mannheim, Germany) as the instructions. Images were captured by FV3000 confocal fluorescence microscope.

### Fluorescence in situ hybridization (FISH)

Cy3-labeled circCYP24A1 (red) probe and FAM-labeled miR-1301-3p (green) probes were purchased from GenePharma. FISH assays were conducted with a FISH Kit (Bersinbio) following the operation manual. DAPI were applied to indicate nuclei. Images were captured by FV3000 confocal fluorescence microscope. The probes are listed in Additional file 11: Table S4.

### Plasmid construction, small interfering RNA (siRNA), lentivirus and cell transfection

For constructing circCYP24A1 plasmids, circCYP24A1 sequence was synthesized and subcloned into pCDH-ciR by GenePharma. CircCYP24A1 siRNAs, ALDH1A3 siRNAs, miR-1301-3p mimics and inhibitor were obtained from GenePharma. SiRNAs and plasmids were transfected with Lipofectamine™ 3000 Reagent (Invitrogen, L3000015). Two circCYP24A1-knockdown lentiviruses, obtained from Genechem (Shanghai, China), were applied to transfect DU145-DR cells. Stable knockdown cells lines were obtained with 6 μg/mL puromycin (YEASEN) selection. All sequences were listed in Additional file 12: Table S5.

### Animal study

Six-week-old male BALB/c athymic nude mice (*n* = 6 per group), purchased from Animal Core Facility of Nanjing Medical University (Nanjing, China), were keep in the specific pathogen-free environment at the Animal Laboratory Center of Nanjing University. Mice were subcutaneously injected with 2 × 10^6^ DU145-DR cells stably transfected with corresponding lentivirus suspended in 100 μL of saline. Two weeks after establishment of mouse model, mice were intraperitoneally administrated with saline or 10 mg/kg DTX every 6 days. Four groups were assigned according to the treatment: group 1^#^, sh-circCYP24A1-transfected cells + DTX; group 2^#^, sh-circ CYP24A1-transfected cells + saline; group 3^#^, sh-NC-transfected cells + DTX; and group 4^#^, sh-NC-transfected cells + saline. Tumor diameters were measured every 3 days, and tumor volume was calculated as length × width^2^ × π / 6. Mice were sacrificed when tumor diameters longer than 1.5 cm. The tumor xenografts were harvested after sacrificed and fixed in paraformaldehyde for subsequent experiments. Animal experiments were approved and performed in accordance with the Institutional Animal Care and Use Committee of Drum Tower Hospital, Medical School of Nanjing University.

### Statistical analysis

Mann-Whitney *U* test was used for continuous variables and the χ^2^ test for categorical variables. Student’s *t* test and one-way analysis of variance (*ANOVA*) were performed to calculate the significance of difference. To predict the chemotherapy response, univariate and multivariate logistic regression analyses were conducted for significant parameters. Receiver operating characteristic (ROC) analysis for the internal discrimination validation was done by SPSS software (IBM, USA). Corresponding area under the curve (AUC) with 95% confidence interval, sensitivity, specificity, and cut-off value for differentiation were derived. Linear correlation analyzes were used to assess correlations between circCYP24A1, miR-1301-3p and ALDH1A3 expression. The software SPSS 26.0 was employed for statistical analysis, of which all tests were two-sided with statistical significance set at *P* < 0.05 (*P < 0.05, **P < 0.01 and ***P < 0,001). All values were shown as the means ± standard deviation of three independent experiments.

## Results

### CircCYP24A1 elevates in DTX-resistant PCa

In order to explore the underlying mechanism of DTX resistance, DTX-resistant cell lines (DU145-DR) were established. Cells were considered to be DTX-resistant when they were able to remain 90–100% viable detected by CCK-8 assay in the presence of this 10-fold higher than IC50 of DTX (5.7 nM), and the IC50 of DU145-DR was 122.3 nM (Additional file 1: Fig. S1A). Moreover, DU145-DR cells showed resistance to DTX in vivo (Additional file 1: Fig. S1B, C). Additionally, flow cytometry analyses revealed that DU145-DR cells had a lower apoptosis rate (Additional file 1: Fig. S1D, F) and less G2/M phrase arrest (Additional file 1: Fig. S1E, G) than the parental cells after treated with 20 nM DTX. In general, DU145-DR cells revealed a significant resistance to DTX compared with the parental DU145 cells in vivo and in vitro.

**Fig. 1:**
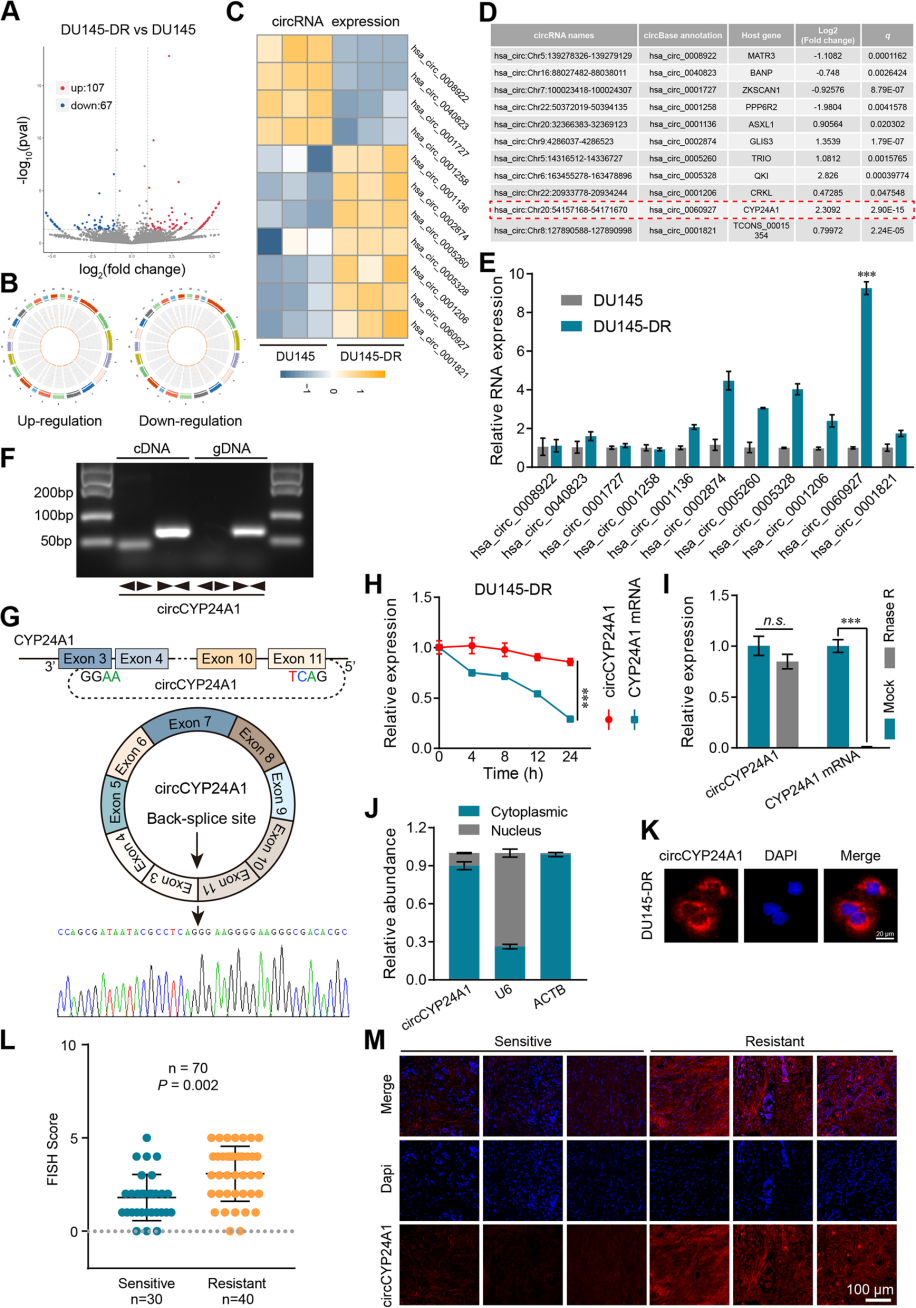
CircCYP24A1 was identified and characterized in DTX-resistant PCa. **A** Volcano plots visualizing 107 upregulated and 67 downregulated circRNAs in DU145-DR cells. **B** The chromosomal localization of those significantly expressed circRNAs. **C** Hot map visualizing 7 upregulated and 4 downregulated circRNAs in DU145-DR cells according to the log_2_ (fold-change) > 1 or < − 1 and *q* value < 0.05. **D** Basic information for the dysregulated circRNAs. **E** qRT-PCR analysis of the dysregulated circRNAs in DU145-DR and DU145 cells. **F** PCR and agarose gel electrophoresis analysis were used to verify the existence of linear and circular forms of CYP24A1 in cDNA and gDNA samples from DU145-DR cells. **G** Schematic illustration of circCYP24A1 back splicing junction validated by Sanger sequencing. **H** ActD treatment was used to evaluate the stability of circCYP24A1 and CYP24A1 mRNA in DU145-DR cells. **I** qRT-PCR analysis of the circCYP24A1 and CYP24A1 mRNA level after RNase R exonuclease treatment. **J** Nuclear-cytoplasmic fractionation assay was performed to observe the subcellular distribution of circCYP24A1 in DU145-DR cells. The ACTB mRNA and U6 snRNA were used as cytoplasmic and nuclear markers, respectively. **K** The circCYP24A1 probe labeled with Cy3 (red) was used to visualize the location of circCYP24A1 in DU145-DR cells via FISH assay, and the nuclei were stained with DAPI (blue). **L** FISH score of circCYP24A1 in DTX-sensitive (*n* = 30) and DTX-resistant (*n* = 40) PCa biopsy tissues. **M** Representative FISH images of circCYP24A1 in three DTX-resistant/−sensitive patients, respectively. The circCYP24A1 probe was labeled with Cy3 (red), and the nuclei were stained with DAPI (blue). All data are presented as the means ± SD of three independent experiments. *n* = 3, ***P < 0.001

RNA sequencing (RNA-seq) analysis was conducted to investigate the differential expression of circRNAs in DTX-resistant DU145 cells. With a qualifying standard of fold-change > 2 (or < − 2) and *P* value < 0.05, 107 upregulated and 67 downregulated circRNAs were identified (Fig. 1A, B; Additional file 2: Fig. S2A). Among these circRNAs, 94.1% were the products from back-splicing of protein-coding exons (Additional file 2: Fig. S2B). In order to amend the “Type I” RNA-seq error and accurately assess the variation, a heat map was generated to visually demonstrate the differential circRNAs expression with false discovery rate < 0.05 (Fig. 1C, D). Finally, 7 upregulated and 4 downregulated circRNAs were further confirmed in DTX-resistant PCa cells using qRT-PCR (Fig. 1E). Among them, has_circ_0060927 (Chr20:54157168–54,171,670) was found to be the most remarkably upregulated in DU145-DR cells. According to the CircBase database annotation, hsa_circ_0060927, with a length of 1106 bp, is back spliced from exons 3 to 11 of *CYP24A1* gene and therefore is named as circCYP24A1. To further confirm the existence of circCYP24A1, the specific convergent and divergent primers were designed respectively. The result revealed that only the amplification from cDNA templates showed the existence of circCYP24A1, while both cDNA and gDNA templates could amplified by convergent primer of the CYP24A1 mRNA (Fig. 1F). Moreover, the back-splicing junction of circCYP24A1 was directly approved by first-generation sequencing analysis (Fig. 1G). Further, ActD and RNase R exonuclease assay were used to confirm the circular form of circCYP24A1, which showed more stable than linear mRNA (Fig. 1H, I). According to the RNA nuclear-cytoplasmic fractionation and FISH assay, circCYP24A1 located predominantly in the cytoplasm (Fig. 1J, K).

Next, we verified the expression of circCYP24A1 in biopsy tissues from DTX sensitive (*n* = 30) and resistant (*n* = 40) patients. Interestingly, the circCYP24A1 expression was found to be significantly higher in DTX-resistant PCa specimens based on the FISH score (*P* = 0.002; Fig. 1L, M; Table 1). These findings revealed that circCYP24A1, elevated in DTX-resistant PCa, might regulate DTX sensitivity.

**Table 1:** Clinical and radiological characteristics of patients with sensitive or resistant to DTX

Characteristics	DTX response status		*p*
	DTX-Resistant (*n* = 40)	DTX-Sensitive (*n* = 30)	
Age (years)	69 (61–74)	66 (63–75)	0.868
PSA pre-treatment (ng/ml)	58.1 (29.6–132.5)	31.6 (17.3–87.1)	0.112
Prostate volume (ml)	17.5 (14.6–20.3)	15.9 (14.5–21.7)	0.577
PSA post-treatment (ng/ml)	0.17 (0.04–1.01)	0.02 (0.01–0.12)	**< 0.001**
Tumor max diameter on MRI	2.6 (1.3–3.4)	2.1 (1.3–2.6)	0.292
PI-RADS			0.514
3	2 (5.0)	3 (10.0)	
4	12 (30.0)	6 (20.0)	
5	26 (65.0)	21 (70.0)	
Clinical T stage			0.360
T1/T2	0 (0)	0 (0)	
T3a	12 (30.0)	14 (46.7)	
T3b	23 (57.5)	13 (43.3)	
T4	5 (12.5)	3 (10.0)	
ISUP grade at biopsy			0.131
1	2 (5.0)	5 (16.7)	
2	9 (22.5)	2 (6.7)	
3	3 (7.5)	5 (16.7)	
4	19 (47.5)	11 (36.7)	
5	7 (17.5)	7 (23.3)	
circCYP24A1 expression			**< 0.001**
Low	14 (35.0)	24 (80.0)	
High	26 (65.0)	6 (20.0)	
miR-1301-3p expression			**0.027**
Low	24 (60.0)	10 (33.3)	
High	16 (40.0)	20 (66.7)	
ALDH1A3 expression			**0.032**
Low	15 (37.5)	19 (63.3)	
High	25 (62.5)	11 (36.7)	

Continuous variables are presented as median (interquartile range, IQR), while categorical variables are presented as patients (%)

*MRI* magnetic resonance imaging, *PSA* prostate-specific antigen, *ISUP* International Society of Urological Pathology, *PI-RADS* Prostate Imaging Reporting and Date System

Significant *P* values were presented in bold text

### CircCYP24A1 promotes DTX resistance of PCa in vitro

**Fig. 2:**
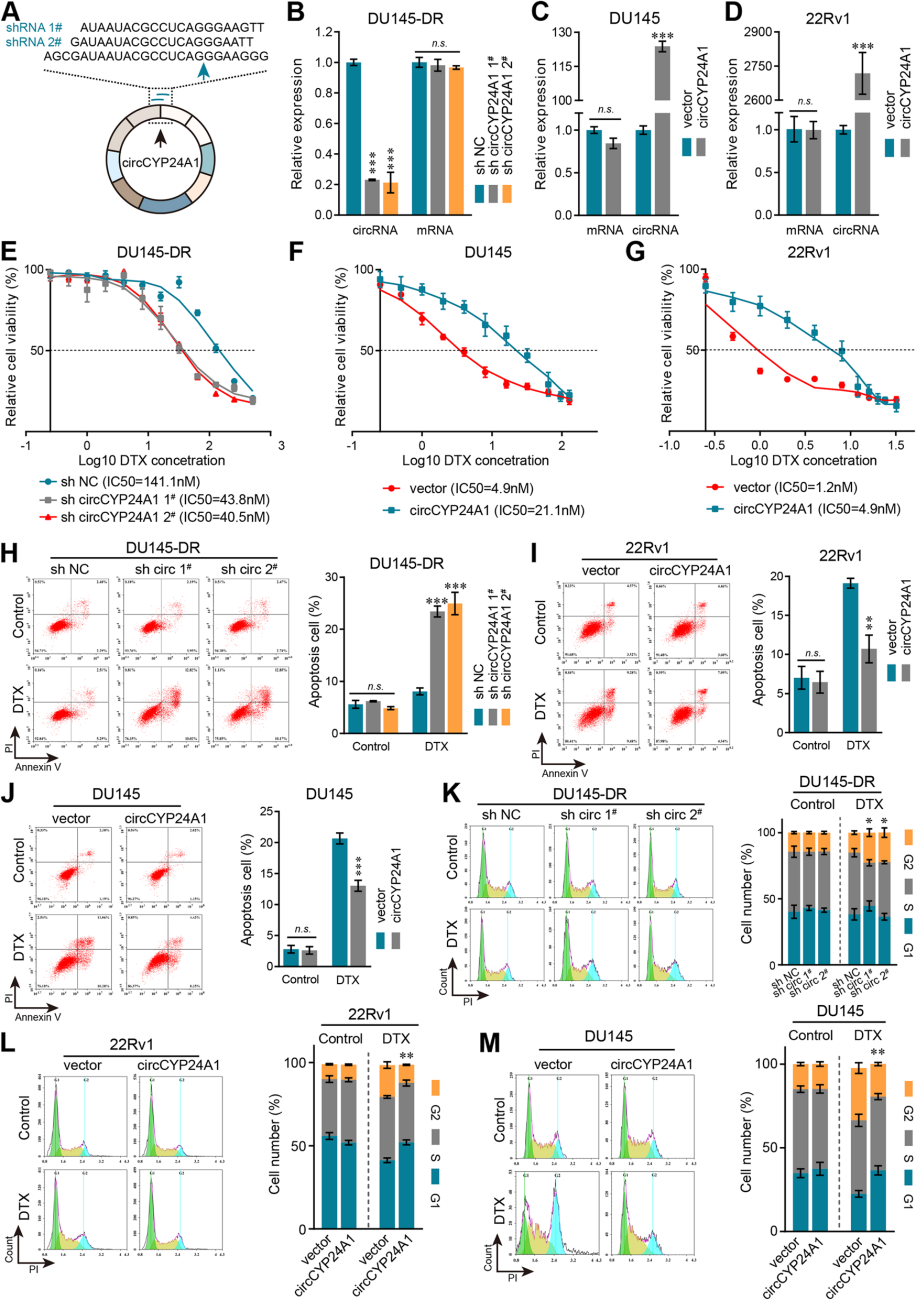
CircCYP24A1 enhances DTX resistance in vitro. **A** Schematic illustration of two sh-circCYP24A1 RNAs targeted the back-splicing junction. **B-D** qRT-PCR analysis of circCYP24A1 and CYP24A1 mRNA expression in DU145-DR (**B**), DU145 (**C**) and 22Rv1 (**D**) cells transfected with indicated lentivirus. **E-G** CCK-8 assays were used to detected the DTX cytotoxicity after down−/up-regulation of circCYP24A1 in DU145-DR (**E**), DU145 (**F**) or 22Rv1 (**G**) cells. **H-J** The indicated cells were treated with DTX (20 nM for DU145-DR/DU145 and 3 nM for 22Rv1) or PBS (control) and subjected to Annexin V-APC and PI stanning to detect apoptotic rate by flow cytometry. **K-M** Cell cycle were detected by flow cytometry in indicated cells after treatment with DTX (20 nM for DU145-DR/DU145 and 3 nM for 22Rv1). All data are presented as the means ± SD of three independent experiments. *n* = 3, *P < 0.05, **P < 0.01, ***P < 0.001

To identify the potential roles of circCYP24A1 in DTX resistance, DU145-DR cells were transfected with shRNAs by lentivirus to knockdown circCYP24A1 (Fig. 2A, B). In addition, circCYP24A1 plasmid were transfected into DU145 and 22Rv1 cell lines for circCYP24A1 overexpression without significantly affecting the expression of CYP24A1 mRNA (Fig. 2C, D). Silence of circCYP24A1 evidently decreased IC50 value of DTX in DU145-DR cells, while the IC50 concentration of DTX was increased in DU145 and 22Rv1 cells with overexpression of circCYP24A1 (Fig. 2E-G). Furthermore, flow cytometry analysis was performed and the results revealed that knockdown circCYP24A1 caused the increased apoptosis rate (Fig. 2H) and more G2/M phrase arrest (Fig. 2K). Correspondingly, overexpression circCYP24A1 reduced cell apoptosis and G2/M phrase arrest under DTX treatment in both DU145 and 22Rv1 cells (Fig. 2I, J, L, M). These findings indicated that circCYP24A1 might partly promote the resistance of PCa cells to DTX.

### CircCYP24A1 suppresses the DTX Chemosensitivity of PCa in vivo

**Fig. 3:**
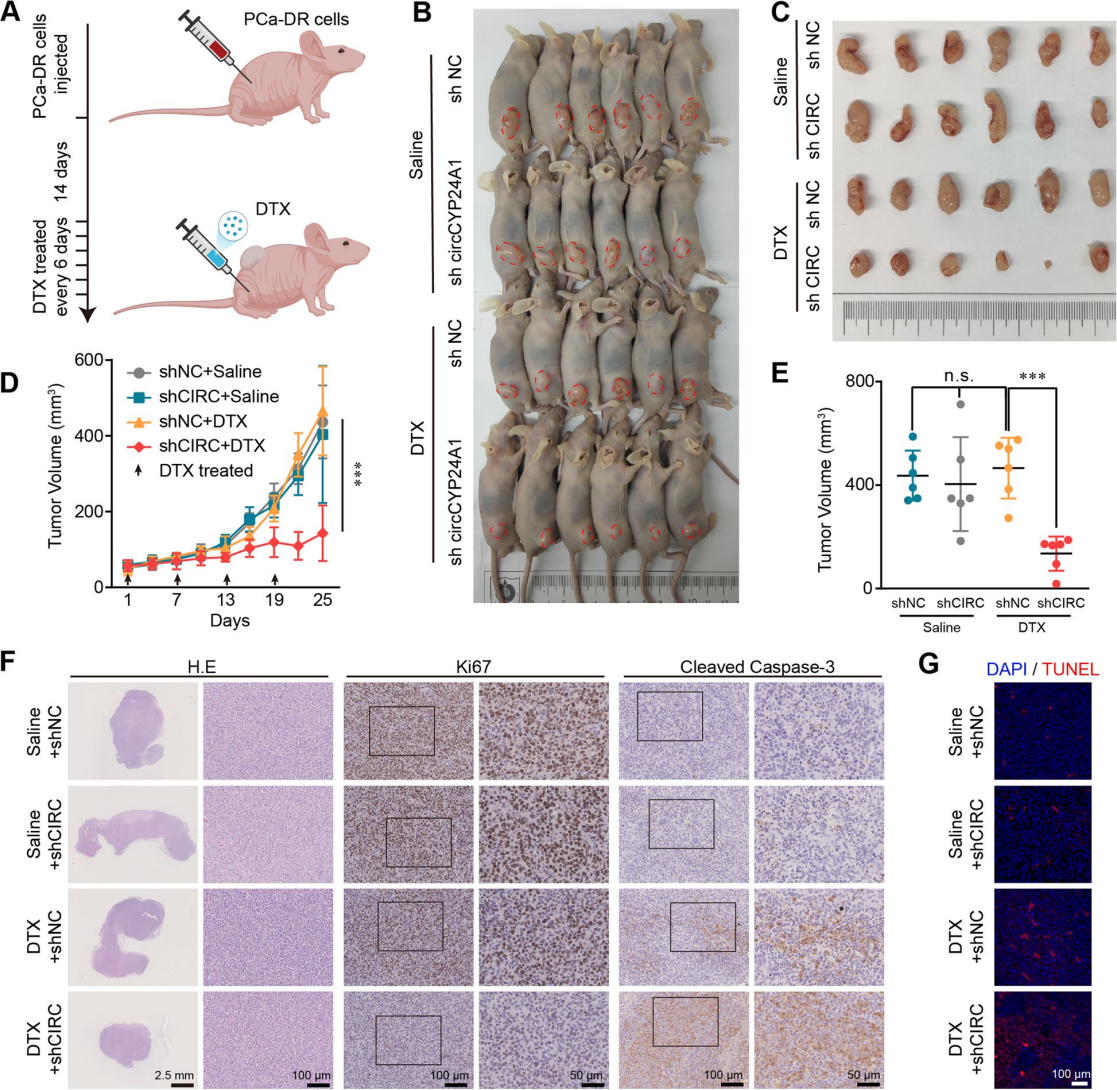
Knocking down circCYP24A1 suppressed the DTX chemoresistance of PCa in vivo. **A** Schematic diagram illustrates the xenograft mouse model treated by indicated concentration of DTX. **B**,** C** Images of the BALB/c nude mice (**B**) and the tumor xenografts (**C**) at the endpoint. **D** Tumor growth curves of tumor sizes are shown. Tumor volumes were measured every 3 days since the first DTX treatment. **E** Tumor volumes measured at the experimental endpoint. **F** Representative images of H&E and IHC (Cleaved caspase 3 and Ki67) staining of the xenograft tumors. **G** TUNEL staining of the xenograft tumors. All data are presented as the means ± SD of three independent experiments. *n* = 6, ***P < 0.001

To further verify the bio-function of circCYP24A1 in DTX chemosensitivity, the mouse xenograft model was established by subcutaneously injected DU145-DR cells transfected with shRNAs of circCYP24A1 into immunodeficient athymic mice. Two weeks after establishment, tumor-bearing nude mice were treated with DTX (10 mg/kg) every 6 days (Fig. 3A). The results revealed that silence of circCYP24A1 increased DTX efficacy, indicated by significantly smaller tumor appearance (Fig. 3B, C). In addition, lower volume of tumors was detected in the DTX/sh-circCYP24A1 group, compared with those in the other groups (Fig. 3D, E). Moreover, significantly increased cleaved caspase 3 expression and decreased Ki67 level were observed in DTX/sh-circCYP24A1 group compared with the DTX/sh-NC group (Fig. 3F). Correspondingly, the DTX/sh-circCYP24A1 group presented more TUNEL-positive nuclei, suggesting more apoptosis after DTX treatment (Fig. 3G). In general, our findings indicated that circCYP24A1 could suppress DTX chemosensitivity in vivo.

### CircCYP24A1 functions as a molecular sponge of miR-1301-3p in PCa cells

**Fig. 4:**
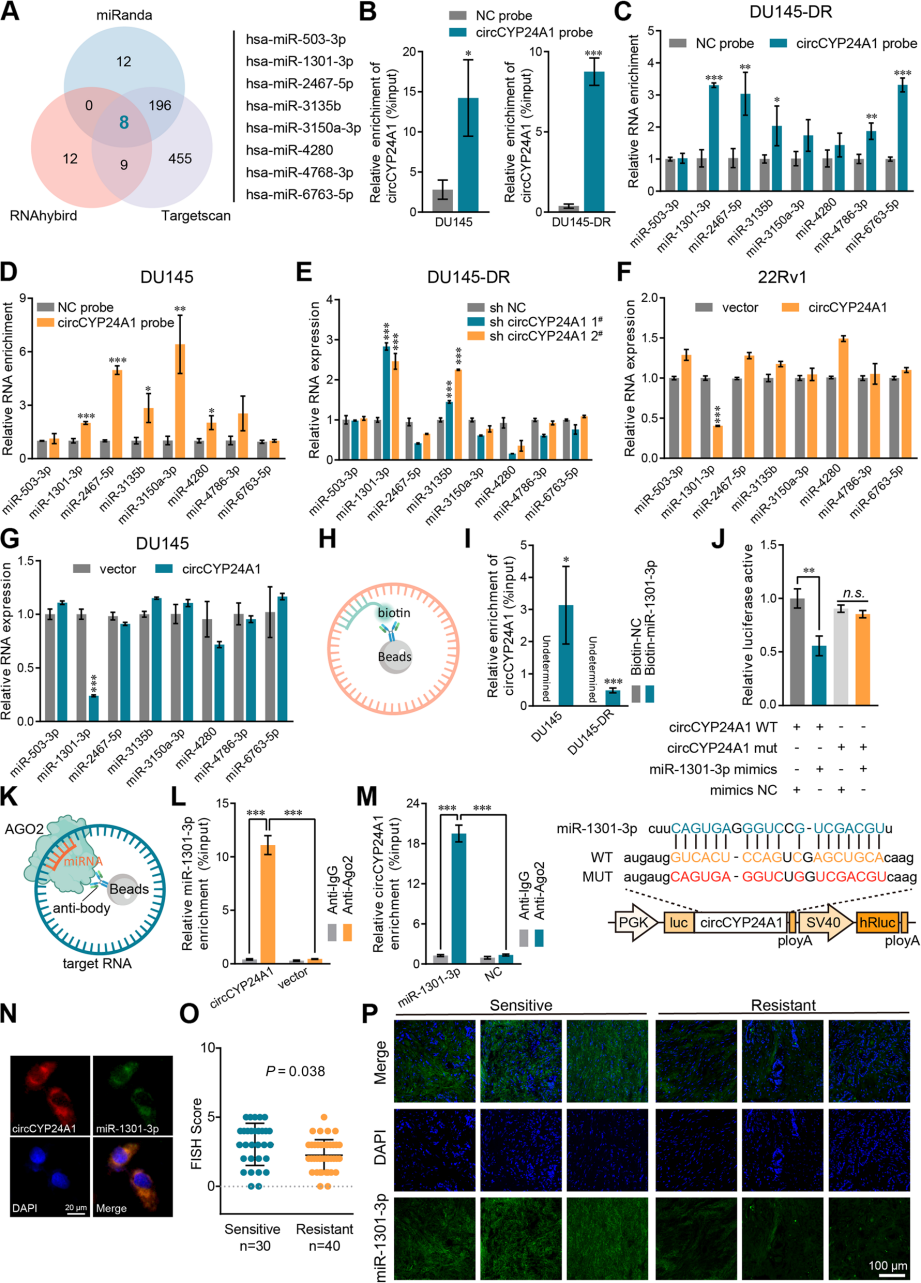
CircCYP24A1 serves as a sponge for miR-1301-1p. **A** Venn diagram showing the overlap of the eight candidate target miRNAs of circCYP24A1 predicted by miRanda, RNAhybird and TargetScan. **B** The efficiency of the circCYP24A1 probe in DU145-DR and DU145 cells. A random sequence probe served as a negative control. **C-D** The fold enrichment of the target miRNAs in pull-down complex of DU145-DR cells (**C**) and DU145 cells (**D**). **E-G** The expression of those candidate miRNAs after down−/up-regulation of circCYP24A1 in DU145-DR (**E**), 22Rv1 (**F**) and DU145 cells **(G)**. H Schematic view of RNA pull down assays by biotin-labeled miRNA. **I** Biotin-labeled miRNA pull-down in DU145 and DU145-DR cells, and qRT-PCR results showing circCYP24A1 enrichment in miRNA probe. A random sequence probe served as a negative control. **J** The luciferase activities of the circCYP24A1 luciferase reporter in HEK293T cells transfected with miR-1301-3p mimics or mimic NC (upper) and the schematic of the wild-type (WT) and mutant (MUT) circCYP24A1 luciferase reporter vectors (bottom). **K** Schematic view of anti-AGO2 RIP assays. **L-M**. Anti-AGO2 RIP assays were performed in DU145 cells, and the enrichment of circCYP24A1 (**L**) and miR-1301-3p (**M**) was detected by qRT-PCR. **N** The colocalization of circCYP24A1 and miR-1301-3p in DU145-DR cells was detected by FISH assay. The circCYP24A1 probe was labeled with Cy3 (red), miR-1301-3p probes were labeled with FAM (green), and nuclei were stained with DAPI (blue). **O** FISH score of miR-1301-3p in DTX-sensitive (*n* = 30) and DTX-resistant (*n* = 40) PCa biopsy tissues. **P** Representative FISH images of miR-1301-3p in three DTX-resistant/−sensitive patients, respectively. The miR-1301-3p probe was labeled with FAM (green), and the nuclei were stained with DAPI (blue). All data are presented as the means ± SD of three independent experiments. *n* = 3, *P < 0.05, **P < 0.01, ***P < 0.001

Growing evidence has shown that lots of circRNAs modulated the downstream mRNA expression by sponging miRNAs [26]. The intracellular distribution of circCYP24A1 suggested the ability of binding miRNAs. The cross-analysis of three miRNA databases (miRanda [27], RNAhybird [28] and Targetscan [29]) was used to predict the possible miRNAs which could bind to circCYP24A1. Eight candidate miRNAs were identified for the further validation (Fig. 4A). Next, the biotin-labeled circCYP24A1 probe was designed to indirectly capture the miRNAs in DU145-DR and DU145 cells. Our results revealed that circCYP24A1 was mostly enriched by the circCYP24A1 probe compared with the random sequence probe, confirming the specificity of the probe (Fig. 4B). Further, miR-2467-5p/− 1301-3p/−3135b were found to be the significantly enriched miRNAs in both DU145-DR and DU145 cells (Fig. 4C, D). Moreover, the levels of miR-1301-3p/−3135b were upregulated in DU145-DR cells after silencing circCYP24A1 (Fig. 4E). Interestingly, circCYP24A1 overexpression only reduced the level of miR-1301-3p in both DU145 and 22Rv1 cells (Fig. 4F, G). In addition, we confirmed that circCYP24A1 had potential effects only on miR-1301-3p expression among those 8 candidate miRNAs by up−/down-regulation of circCYP24A1. Furthermore, the RNA pull-down assays verified the interaction between biotin-labelled miR-1301-3p and circCYP24A1 (Fig. 4H, I). Dual-luciferase assay was used to confirm that miR-1301-3p could bind to circCYP24A1 directly. The full length of circCYP24A1 (wild-type, WT) was subcloned into pGLO-miR, and the mutation plasmid containing mutated potential binding region of miR-1301-3p was constructed. Luciferase activities were measured after transfected with miR-1301-3p and luciferase reporter plasmid, and a remarkable reduction of luciferase activities could be abolished by mutation (Fig. 4J). As previously reported, AGO2 was characterized as a potent component of RNA-induced silencing complex to participate the function of miRNA [30]. Thus, an anti-AGO2 RIP assay was performed to further testify the interaction between circCYP24A1 and miR-1301-3p (Fig. 4K). The ectopic expression of circCYP24A1 notably increased the enrichment of miR-1301-3p in AGO2 complex instead of IgG (Fig. 4L). Similarly, upregulation of miR-1301-3p dramatically increased enrichment of circCYP24A1, compared with that in the negative control (Fig. 4M). FISH assays visualized that circCYP24A1 and miR-1301-3p colocalized in the cytosol of DU145-DR cells (Fig. 4N). Moreover, miR-1301-3p levels were assessed by FISH score. Higher level of miR-1301-3p was found in specimen from those DTX-sensitive patients, compared with those from DTX-resistant patients (Fig. 4O, P; Table 1). In summary, these results suggested that miR-1301-3p might directly interact with circCYP24A1 in potential binding sites.

### CircCYP24A1 enhances the DTX-resistance of PCa cells through miR-1301-3p

**Fig. 5:**
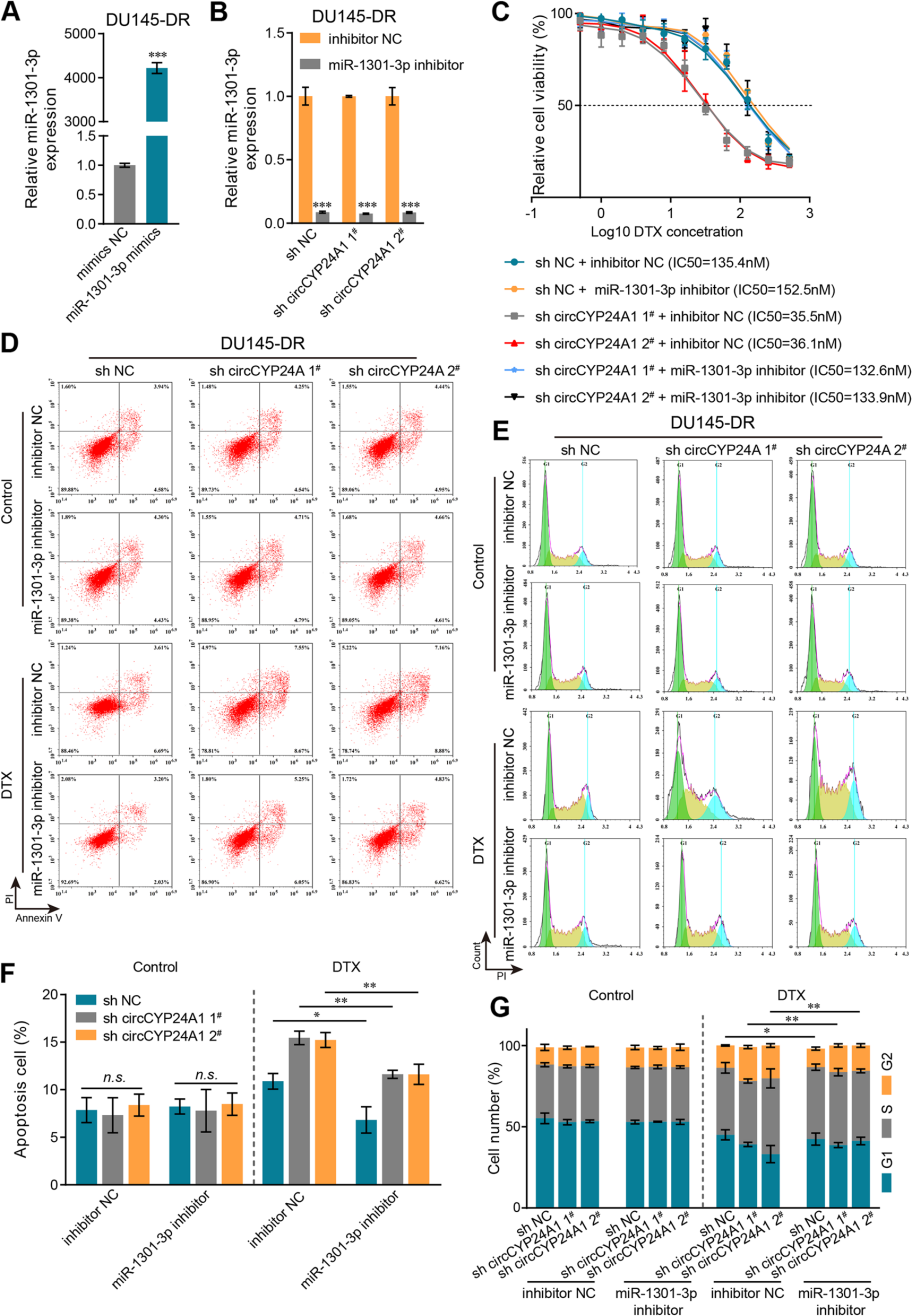
CircCYP24A1 sponges miR-1301-3p to regulate DTX sensitivity in PCa cells. **A** The expression of miR-1301-3p in DU145-DR cells transfected with miR-1301-3p mimics. **B** The expression of miR-1301-3p in DU145-DR cells transfected with shcircCYP24A1 and miR-1301-3p inhibitor. **C** CCK-8 data for the IC50 of DU145-DR cells treated with 20 nM DTX after transfection with miR-1301-3p inhibitor combined with or without sh-circCYP24A1. **D-E** The indicated cells were treated with 20 nM DTX and subjected to detect apoptotic rate (**D**) or cell cycle (**E**) by flow cytometry. **F** Statistical analysis of the apoptotic rate. **G** Statistical analysis of the cell cycle. All data are presented as the means ± SD of three independent experiments. *n* = 3, *P < 0.05, **P < 0.01, ***P < 0.001

To further verify the bio-functions of miR-1301-3p in DTX-resistant, corresponding mimics and inhibitor were used to regulate its expression in DU145-DR cells. The efficiency of miR-1301-3p mimics and inhibitor in cells was examined by qRT-PCR (Fig. 5A, B). The results of CCK-8 assays indicated that the miR-1301-3p inhibitor substantially attenuated DTX cytotoxicity and reversed the reduced IC50 value caused by circCYP24A1 downregulation (Fig. 5C). Similarly, flow cytometry analysis revealed that miR-1301-3p inhibitor could markedly decrease apoptosis rate (Fig. 5D, F) and less G2/M phrase arrest (Fig. 5E, G). Collectively, these data illustrated that miR-1301-3p promoted DTX sensitivity in DU145-DR cells and played a crucial function in the downstream of circCYP24A1.

### CircCYP24A1 upregulates ALDH1A3 expression by post-transcriptional mechanisms in DTX-resistant PCa cells

**Fig. 6:**
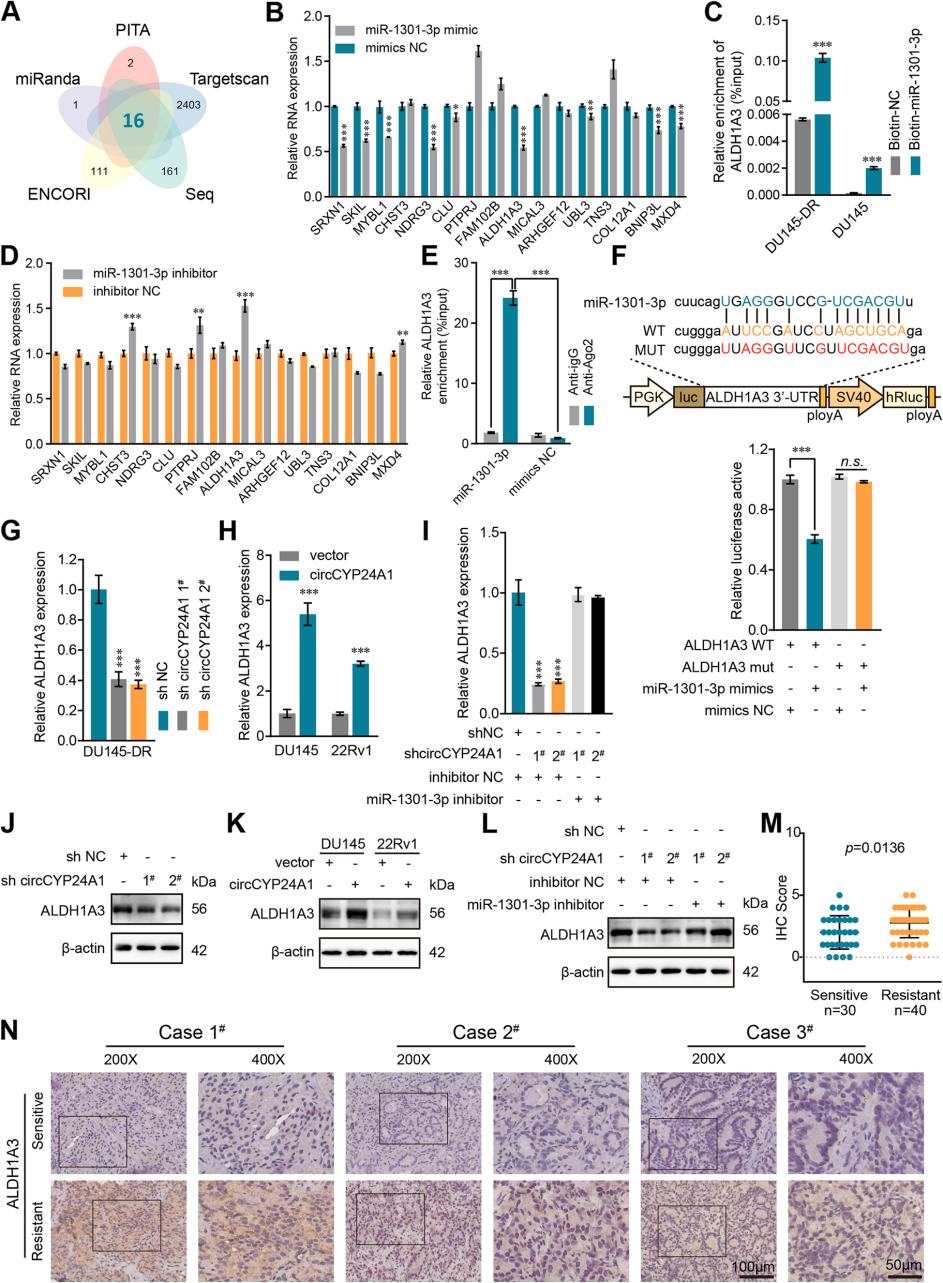
CircCYP24A1 upregulates ALDH1A3 expression by post-transcriptional mechanisms in DTX-resistant PCa. **A** Venn diagram visualizing the overlap of 13 genes which was predicted according to the cross-analysis of PITA, miRanda, ENCORI, TargetScan and the mRNA-seq results. **B, D** The expression of those candidate genes after transfected with miR-1301-3p mimic (**B**) or inhibitor (**D**). **C** The enrichment of ALDH1A3 mRNA was detected by biotinylated miRNA pull-down in DU145-DR and DU145 cells. **E** Anti-AGO2 RIP assays were performed in DU145 cells, and the enrichment of ALDH1A3 was assessed by qRT-PCR. **F** The luciferase activities were determined in HEK293T cells co-transfected with ALDH1A3 luciferase reporter vectors and miR-1301-3p mimics or mimic NC (bottom), and the schematic showed wild-type (WT) and mutant (MUT) ALDH1A3 luciferase reporter vectors (upper). **G-L** The RNA and protein levels of ALDH1A3 was detected by qRT-PCR or western blot in indicated cells, respectively. **M** IHC score of ALDH1A3 in DTX-sensitive (*n* = 30) and DTX-resistant (*n* = 40) PCa biopsy tissues. **N** Representative IHC images of ALDH1A3 in three DTX-resistant/−sensitive patients, respectively. All data are presented as the means ± SD of three independent experiments. *n* = 3, **P < 0.01. ***P < 0.001

Cross-analysis between four bioinformatic database (PITA, miRanda, Targetscan and ENCORI) and mRNA-seq upregulated in DU145-DR cells were performed to explore the function of miR-1301-3p in DTX-resistant PCa cells. As the results, 16 potential target genes of miR-1301-3p were identified for the following investigations (Fig. 6A). ALDH1A3 was found to be the most possible downstream target of miR-1301-3p according to significant regulation of ALDH1A3 by miR-1301-3p (Fig. 6B, D). Thus, we supposed that ALDH1A3 and miR-1301-3p might form a ceRNA network in DTX-resistant PCa.

To test the above hypothesis, biotin-labelled miR-1301-3p based RNA pull-down assays and anti-AGO2 RIP assays were performed to verify the direct interaction between miR-1301-3p and ALDH1A3 mRNA. As shown in Fig. 6C, biotin-labelled miR-1301-3p could directly pull down ALDH1A3 mRNA in both DU145-DR and DU145 cells. Likewise, the overexpression of miR-1301-3p augmented the enrichment of ALDH1A3 mRNA in AGO2 (Fig. 6E). Further, the wild-type and mutant ALDH1A3 3′-UTR were cloned into dual-luciferase reporter plasmids respectively. The luciferase activity was notably downregulated after ectopic expression of miR-1301-3p, which showed no influence on MUT-3′-UTR of ALDH1A3 plasmid (Fig. 6F). Moreover, down−/up- regulation of circCYP24A1 could modulate the mRNA and protein levels of ALDH1A3, which could be mostly rescued while silencing miR-1301-3p (Fig. 6G-L; Additional file 3: Fig. S3). The IHC staining results revealed that ALDH1A3 were upregulated induced DTX treatment and downregulated due to circCYP24A1 knockdown in tumor-bearing mice (Additional file 4: Fig. S4). Similar as circCYP24A1, the IHC score emphasized that the expression of ALDH1A3 were evidently elevated in DTX-resistant PCa patients compare with those sensitive to DTX (Fig. 6M, N). To summarize, the enhanced expression of ALDH1A3 was induced by circCYP24A1 via regulation of miR-1301-3p through post-transcriptional mechanisms and related to DTX resistance.

### ALDH1A3 regulates the influence of circCYP24A1 on the DTX chemosensitivity of PCa cells via PI3K/AKT/mTOR axis

**Fig. 7:**
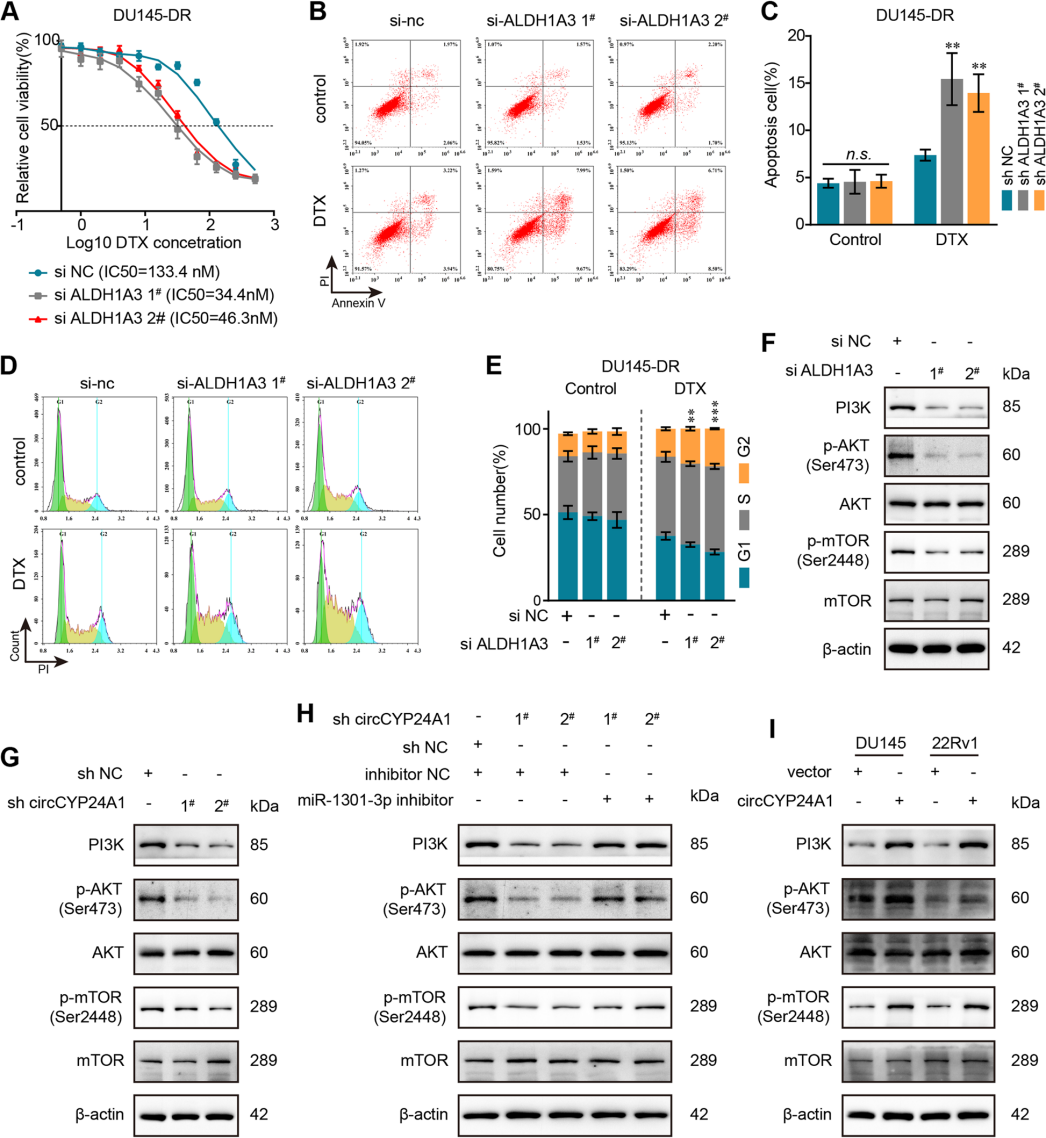
ALDH1A3 promotes DTX resistance via activating PI3K/AKT/mTOR signaling pathway. **A** The IC50 values of DU145-DR cells were detected after treatment with 20 nM DTX and transfection with si-ALDH1A3. **B-E** Flow cytometry analysis were used to assess cell apoptosis (**B, C**) and cell cycle (**D, E**) in DU145-DR cells treated with 20 nM DTX after transfection of si-ALDH1A3. **F-I** The protein levels of PI3K and downstream molecules were determined in those cells co-transfected with siRNAs or overexpression plasmid and miR-1301-3p inhibitors by western blot. All data are presented as the means ± SD of three independent experiments. *n* = 3, **P < 0.01, ***P < 0.001

In the previous studies, ALDH1A3 has been verified to play a fundamental role in resistance of temozolomide [31]. Nonetheless, there has been none evidence supporting the association of ALDH1A3 and DTX resistance. To further confirm whether ALDH1A3 is involved in DTX resistance, cell cytotoxicity and IC50 assays were performed to verify this hypothesis. We found that knockdown of ALDH1A3, validated by western blot (Additional file 5: Fig. S5), induced decreased IC50 and mitigated DTX resistance in DU145-DR cells (Fig. 7A). Moreover, flow cytometry analysis uncovered that a notably increased apoptosis rate was induced by si-ALDH1A3 under the treatment of DTX in DU145-DR cells (Fig. 7B, C), and more G2/M phrase arrest was observed correspondingly (Fig. 7D, E).

In accordance, PI3K/AKT signal pathway was reported to function momentously in various tumors to induce the resistance of CDK4/6 inhibitors or temozolomide and be involved in ALDH1A3-dependent tumor progression [32–34]. Obviously, our results illustrated that silence of ALDH1A3 induced a notably depressed activity of PI3K/AKT/mTOR pathway in DU145-DR cells (Fig. 7F). Likewise, knockdown of circCYP24A1 inactivated this pathway, which could be rescued by the knockdown of miR-1301-3p (Fig. 7G, H). In addition, we found that overexpressed circCYP24A1 enhanced the activity of PI3K/AKT/mTOR pathway (Fig. 7I). According to these findings, ectopic expression of ALDH1A3 might induce DTX resistance in PCa via PI3K/AKT/mTOR signaling pathway.

The clinical characteristics of the 70 included patients were listed in Additional file 13: Table S6, and the biological characteristics were based on the score of IHC standing (ALDH1A3) and FISH assay (circCYP24A1 and miR-1301-3p). Scatter plots illustrated a close relationship between the expression of circCYP24A1, miR-1301-3p and ALDH1A3, respectively (*P* < 0.05) (Additional file 6: Fig. S6 A-C). Meanwhile, the expression of circCYP24A1, miR-1301-3p and ALDH1A3 as well as the post-treatment prostate specific antigen (PSA) level were significantly associated with DTX chemotherapy response (*P* < 0.05 Table 1). In multivariable logistic regression analyses, both the expression of circCYP24A1 (OR = 0.165; 95% CI: 0.038–0.723; *P* = 0.017) and post-treatment PSA (OR = 0.033; 95% CI: 0.002–0.609; *P* = 0.022) could serve as independent risk factors of DTX response (Table 2, Additional file 7: Fig. S7,). In summary, these findings revealed that circCYP24A1, miR-1301-3p and ALDH1A3 were significantly associated with DTX resistance, and circCYP24A1 could be used to predict DTX response.

**Table 2:** Univariate and multivariate logistic regression analyses of clinical and biological parameters for the prediction of DTX response after chemotherapy

Parameters	Univariable logistic regression			Multivariable logistic regression		
	OR	95% CI	*P*	OR	95% CI	*P*
Age (years)	0.993	0.931–1.059	0.830			
PSA pre-treatment (ng/ml)	0.999	0.994–1.004	0.596			
Prostate volume (ml)	0.965	0.890–1.047	0.395			
PSA post-treatment (ng/ml)	0.072	0.007–0.719	**0.025**	0.033	0.002–0.609	**0.022**
Tumor max diameter on MRI	0.792	0.504–1.244	0.311			
PI-RADS						
4 vs. 3	0.333	0.043–2.564	0.291			
5 vs. 3	0.538	0.082–3.526	0.519			
Clinical T stage						
T3a vs. T4	1.944	0.383–9.883	0.423			
T3b vs. T4	0.942	0.193–4.595	0.941			
ISUP grade at biopsy						
2 vs 1	0.089	0.009–0.839	**0.035**	0.137	0.007–2.824	0.198
3 vs 1	0.667	0.076–5.878	0.715	0.538	0.027–10.591	0.684
4 vs 1	0.232	0.038–1.401	0.111	0.331	0.026–4.205	0.394
5 vs 1	0.400	0.057–2.800	0.356	0.423	0.026–6.750	0.542
circCYP24A1 expression						
Low vs High	0.135	0.045–0.407	**< 0.001**	0.165	0.038–0.723	**0.017**
miR-1301-3p expression						
Low vs High	3.000	1.117–8.058	**0.029**	1.096	0.252–4.766	0.903
ALDH1A3 expression						
Low vs High	0.347	0.130–0.926	**0.035**	0.426	0.099–1.832	0.251

Significant *P* values were presented in bold text

*MRI* magnetic resonance imaging, *PSA* prostate specific antigen, *ISUP* International Society of Urological Pathology, *PI-RADS* Prostate Imaging Reporting and Date System, *OR* Odds ratio, *CI* confidence intervals

## Discussion

DTX-based chemotherapy is recommended as the first-line therapy for mCRPC patients [3]. Since the publication of STAMPEDE and CHAARTED trials, DTX-mediated chemotherapy has become the first-line treatment for patients with mHSPC [5]. Even though DTX significantly prolongs the lifespan and improves the clinical outcomes, most patients develop DTX-resistance within 3 years [35]. Several molecular mechanisms regarding DTX resistance has been proposed. CircRNAs, a novel class of ncRNAs, had been suggested to mediate chemotherapy-resistance in multiple cancer. For instance, circRNA-SORE increases sorafenib resistance by stabilizing YBX1 in hepatocellular carcinoma [22]. CircAKT3 enhances resistance of cisplatin via miR-198/PI3KR1 axis in gastric cancer [36]. In present study, we identified that circCYP24A1 was upregulated in DU145-DR cells and DTX-resistant PCa tissues. Further experimental results indicated that circCYP24A1 promoted DTX resistance, suggesting that circCYP24A1 could serve as a potential DTX-resistant biomarker in PCa patients.

Recently, emerging evidence has indicated that the most common biological function of circRNAs was to play as molecular sponges of miRNAs and form ceRNA network to involve post-transcriptional regulation [37]. In this study, we found that circCYP24A1 could provide multiple potential binding sites for eight miRNAs according to the cross-analysis of miRNA databases. Subsequently, the data of RNA pull-down and RIP assays uncovered that miR-1301-3p was the most highly enriched candidate at circCYP24A1. Moreover, the rescue experiment revealed that suppression of DTX resistance induced by circCYP24A1-knockdown could be rescued mostly by miR-1301-3p inhibitor. Based on the above results, we confirmed that circCYP24A1 sponged miR-1301-3p and formed an activated ceRNA regulatory network to induce DTX-resistant in PCa.

It has been well described that, miRNAs exerted their biological functions mostly through post-transcriptional regulation by suppressing the translation or promoting the degradation of target mRNA. Khalid et al. reported miR-138 enhanced DTX resistance via K2/β-integrin axis in mCRPC [38] while Xu et al. showed miR-143 promoted DTX cytotoxicity through suppression of KRAS [39]. Several previous studies have suggested the anti-tumor activity of miR-1301-3p. Qiao et al. verified that miR-1301-3p suppressed tumor development of thyroid papillary cancer through decreasing expression of PCNA [40]. Peng et al. illustrated miR-1301-3p inhibited tumor proliferation through directly mediating ICT1 mRNA in breast cancer [41]. Nevertheless, there has been none evidence to verify the bio-functions of miR-1301-3p in DTX resistance. According to our results, ALDH1A3 and MXD4 were characterized as the potential target mRNA of miR-1301-3p. Meanwhile, we observed that ALDH1A3 mRNA levels had a higher fold-change than MDX4 after transfection of miR-1301-3p mimics or inhibitor, thus we set ALDH1A3 as the target mRNA for further investigation. Here, the results revealed that miR-1301-3p could directly interact with the ALDH1A3 mRNA and downregulated ALDH1A3 in DTX-resistant PCa.

ALDH1A3, a member of aldehyde dehydrogenase family 1, has been proven to convert acetaldehyde to acetate to produce pyruvate [42], nuclear acetyl-CoA [43, 44] and citrate [45]. Interestingly, few studies illustrated the connection between ALDH1A3 expression and DTX resistance. It has been reported that LoVo-1 and K1 cells with high expression of ALDH1A3 were resistant to the focal adhesion kinase autophosphorylation inhibitor Y15, while cancer cells with low ALDH1A3 level showed high sensitivity to Y15 [46]. A study raised by Kong. et al. displayed the pleural malignant mesotheliomas with dysregulation of ALDH1A3 were robustly resistant to cisplatin and pemetrexed [47]. In our study, a sequence of functional experiments data suggested that knockdown of ALDH1A3 decreased the resistance of DTX and increased the DTX-induced apoptosis. Moreover, our findings suggested that the abnormal expression of ALDH1A3 promoted DTX resistance via PI3K/AKT/mTOR axis.

From the multivariable analyses, the expression of circCYP24A1 and post-treatment PSA were found to be independent risk factors of DTX response. Though magnetic resonance imaging (MRI) is the standard imaging for detecting significant PCa, the parameters on MRI did not show good predicting performance compared with biomarkers such circRNA and PSA. As we know, PSA is the most common biomarker for the diagnosis of PCa. Also, post-treatment PSA has been reported to be effective to predict treatment response of neoadjuvant therapy [48, 49]. Zilli, T. et al. illustrated that nadir PSA before radiotherapy was a vitally independent risk factor for the prediction of the treatment response to neoadjuvant ADT [50]. However, nadir PSA could only be revealed after several cycles of treatment, which was not a valuable biomarker to determine the initial treatment strategies. Of interest, circCYP24A1 could be measured before the treatment, suggesting it to be a useful biomarker for decision making of the treatment options.

## Conclusions

**Fig. 8:**
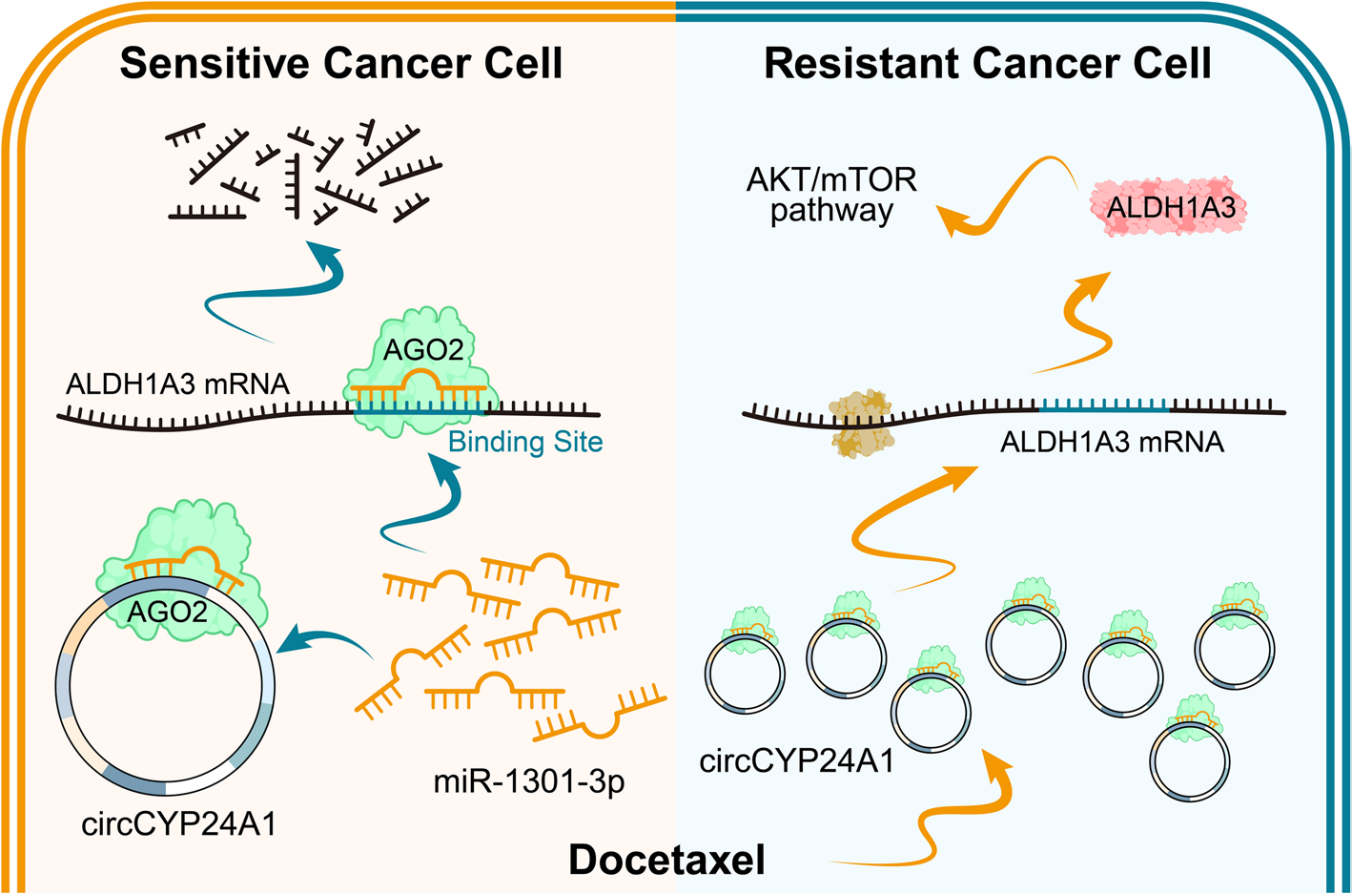
Schematic diagram for the mechanisms of circCYP24A1 functioning as a miRNA sponge to promote DTX resistance in PCa through circCYP24A1/miR-1301-3p/ALDH1A3/PI3K/AKT/mTOR signaling pathway

Our findings illustrated that circCYP24A participated in the generation and maintenance of DTX resistance potently, suggesting that circCYP24A1 could function as a crucial and promising biomarker to predict DTX treatment response and a therapeutic target in patients who are resistant to DTX chemotherapy (Fig. 8).

## Supplementary Information

**Additional file 1: Figure S1.** DTX-resistant cell lines (DU145-DR) were established. A. CCK-8 assays were performed to detected the DTX cytotoxicity in DU145 and DU145-DR cells. B. Tumor growth curves of tumor sizes are shown. Tumor volumes were measured every 3 days since the first DTX treatment. C. The weight growth curves of every group mouse. Mice weight were measured every 3 days during treatment. D, F. DU145 and DU145-DR cells were treated with DTX (20 nM) or PBS (control) and subjected to Annexin V-APC and PI stanning to detect apoptotic rate by flow cytometry. E, G. Cell cycle were detected by flow cytometry in indicated cells after treatment with DTX (20 nM). All data are presented as the means ± SD of three independent experiments. ***P < 0.001.

**Additional file 2: Figure S2.** RNA sequencing analysis were conducted to investigate the differential expression of circRNAs in DTX-resistant DU145 cells. A. Hot map visualizing 107 upregulated and 67 downregulated circRNAs in DU145-DR cells according to the log2 (fold-change) > 1 or < − 1 and *P* value < 0.05. B. Pie chart showed the type of those abnormally expressed circRNAs.

**Additional file 3: Figure S3.** The statistic diagram showed the quantification of the band intensity of ALDH1A3 protein level in in indicated cells. A. ALDH1A3 protein level after knocking down circCYP24A1 expression in DU145-DR cells. B. ALDH1A3 protein level after upregulated circCYP24A1 expression in DU145 and 22RV1 cells. C. ALDH1A3 protein level after transfected with shcircCYP24A1 and miR1301-3p inhibitor in DU145-DR cells.

**Additional file 4: Figure S4.** Representative images of IHC (ALDH1A3) staining of the xenograft tumors.

**Additional file 5: Figure S5.** Two ALDH1A3 siRNAs were used to knock-down ALDH1A3 expression. Western blot was performed to validate si-ALDH1A3 efficiency.

**Additional file 6: Figure S6.** Scatter plot shows the correlation among the level of circCYP24A1, miR-1301-3p and ALDH1A3. A. CircCYP24A1 with miR-1301-3p. (R = − 0.45, *P* < 0.001) B. circCYP24A1 with ALDH1A3. (R = 0.39, *P* < 0.001) C. MiR-1301-3p with ALDH1A3. (R = − 0.33, *P* = 0.0047).

**Additional file 7: Figure S7.** ROC analysis to compare the diagnostic accuracy of the identified parameters. PSA = prostate specific antigen; AUC = area under the curve; 95% confidence intervals is labeled in parentheses.

**Additional file 8: Table S1.** Primers used for real-time PCR.

**Additional file 9: Table S2.** Probes used for RNA pull-down.

**Additional file 10: Table S3.** Primary antibodies used in this study.

**Additional file 11: Table S4.** Probes used for FISH assay.

**Additional file 12: Table S5.** SiRNA used for silencing target genes.

**Additional file 13: Table S6.** Baseline characteristics of the included 70 high-risk PCa patients who received neoadjuvant therapy with DTX.

## Data Availability

The datasets used and/or analyzed during the current study are available from the corresponding author on reasonable request.

## Abbreviations

PCa: Prostate cancer
DTX: Docetaxel
mHPSC: Metastatic hormone-sensitive prostate cancer
mCRPC: Metastatic castration-resistant prostate cancer
ncRNAs: Noncoding RNAs
circRNAs: Circular RNAs
miRNAs: MicroRNAs
siRNAs: Small interfering RNAs
cDNA: Complementary DNA
HRP: Horseradish peroxidase
qRT-PCR: Quantitative realtime PCR
CCK8: Cell Counting Kit 8
TUNEL: TdT-mediated dUTP nick end labeling
RIP: RNA immunoprecipitation
IHC: Immunohistochemistry
PI: Propidium iodide
FISH: Fluorescence in situ hybridization
ROC: Receiver operating characteristic
AUC: Area under the curve
ceRNA: Competing endogenous RNA
MRI: Magnetic resonance imaging
pCR: Pathologic complete response
MDR: Minimal residual disease
