# Supplementary material for: circCYP24A1 promotes Docetaxel resistance in prostate Cancer by Upregulating ALDH1A3
Source: Biomark Res. 2022 Jul 13;10:48. doi: 10.1186/s40364-022-00393-1 (PMC9277795; doi:10.1186/s40364-022-00393-1)
Supplement: Supplementary file 1 — Additional file 1: Figure S1. DTX-resistant cell lines (DU145-DR) were established. A. CCK-8 assays were performed to detected the DTX cytotoxicity in DU145 and DU145-DR cells. B. Tumor growth curves of tumor sizes are shown. Tumor volumes were measured every 3 days since the first DTX treatment. C. The weight growth curves of every group mouse. Mice weight were measured every 3 days during treatment. D, F. DU145 and DU145-DR cells were treated with DTX (20 nM) or PBS (control) and subjected to Annexin V-APC and PI stanning to detect apoptotic rate by flow cytometry. E, G. Cell cycle were detected by flow cytometry in indicated cells after treatment with DTX (20 nM). All data are presented as the means ± SD of three independent experiments. ***P < 0.001. [file 40364_2022_393_MOESM1_ESM.docx]

**Additional file 1: Figure S1**


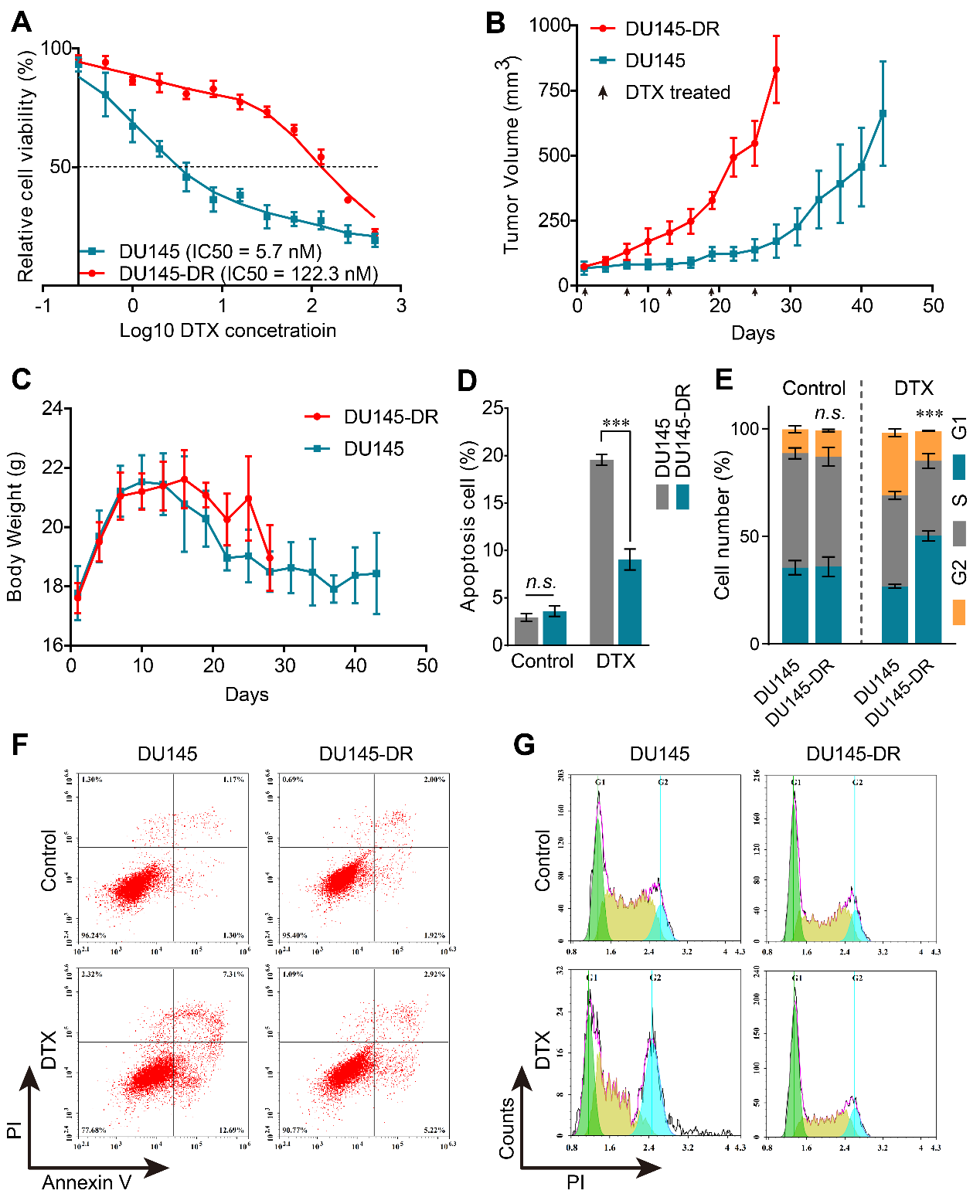


**Figure S1. DTX-resistant cell lines (DU145-DR) were established. A.** CCK-8 assays were performed to detected the DTX cytotoxicity in DU145 and DU145-DR cells. **B.** Tumor growth curves of tumor sizes are shown. Tumor volumes were measured every 3 days since the first DTX treatment. **C.** The weight growth curves of every group mouse. Mice weight were measured every 3 days during treatment. **D, F.** DU145 and DU145-DR cells were treated with DTX (20nM) or PBS (control) and subjected to Annexin V-APC and PI stanning to detect apoptotic rate by flow cytometry. **E, G.** Cell cycle were detected by flow cytometry in indicated cells after treatment with DTX (20 nM). All data are presented as the means ± SD of three independent experiments. ****P* < 0.001
