# Supplementary material for: circCYP24A1 promotes Docetaxel resistance in prostate Cancer by Upregulating ALDH1A3
Source: Biomark Res. 2022 Jul 13;10:48. doi: 10.1186/s40364-022-00393-1 (PMC9277795; doi:10.1186/s40364-022-00393-1)
Supplement: Supplementary file 2 — Additional file 2: Figure S2. RNA sequencing analysis were conducted to investigate the differential expression of circRNAs in DTX-resistant DU145 cells. A. Hot map visualizing 107 upregulated and 67 downregulated circRNAs in DU145-DR cells according to the log2 (fold-change) > 1 or < − 1 and P value < 0.05. B. Pie chart showed the type of those abnormally expressed circRNAs. [file 40364_2022_393_MOESM2_ESM.docx]

**Additional file 2: Figure S2**


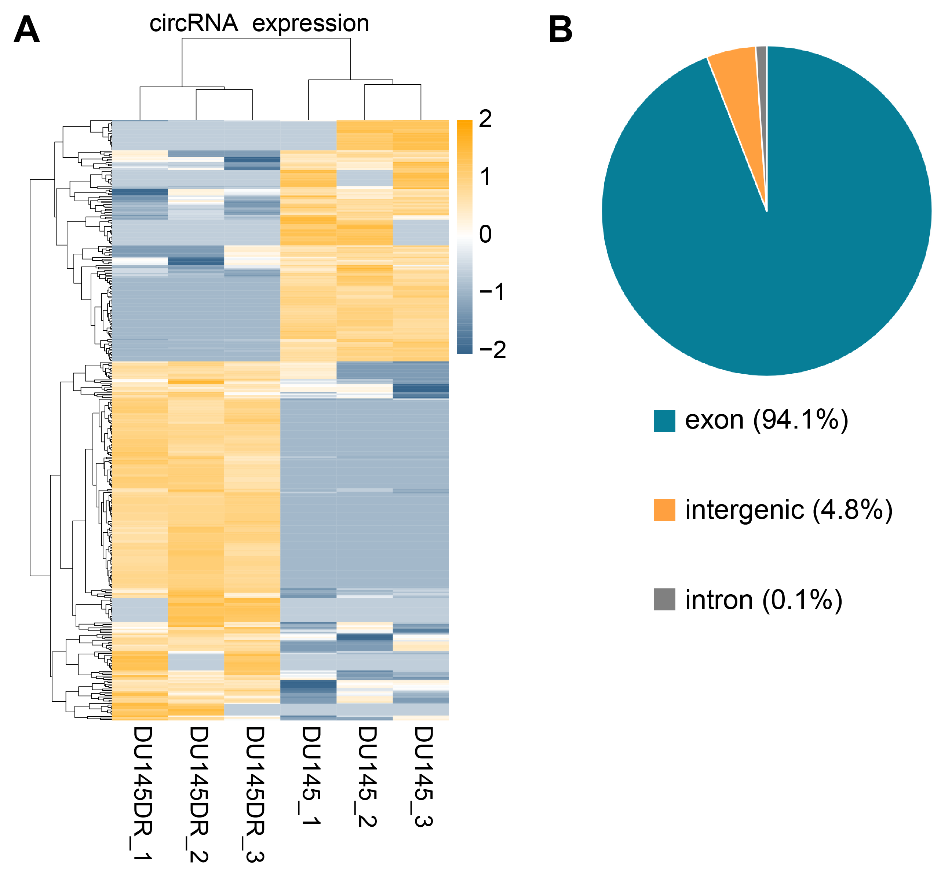


**Figure S2. RNA sequencing analysis were conducted to investigate the differential expression of circRNAs in DTX-resistant DU145 cells. A.**Hot map visualizing 107 upregulated and 67 downregulated circRNAs in DU145-DR cells according to the log2 (fold-change) > 1 or < -1 and *P* value < 0.05. **B.** Pie chart showed the type of those abnormally expressed circRNAs.
