# Supplementary material for: circCYP24A1 promotes Docetaxel resistance in prostate Cancer by Upregulating ALDH1A3
Source: Biomark Res. 2022 Jul 13;10:48. doi: 10.1186/s40364-022-00393-1 (PMC9277795; doi:10.1186/s40364-022-00393-1)
Supplement: Supplementary file 3 — Additional file 3: Figure S3. The statistic diagram showed the quantification of the band intensity of ALDH1A3 protein level in in indicated cells. A. ALDH1A3 protein level after knocking down circCYP24A1 expression in DU145-DR cells. B. ALDH1A3 protein level after upregulated circCYP24A1 expression in DU145 and 22RV1 cells. C. ALDH1A3 protein level after transfected with shcircCYP24A1 and miR1301-3p inhibitor in DU145-DR cells. [file 40364_2022_393_MOESM3_ESM.docx]

**Additional file 3: Figure S3**

**
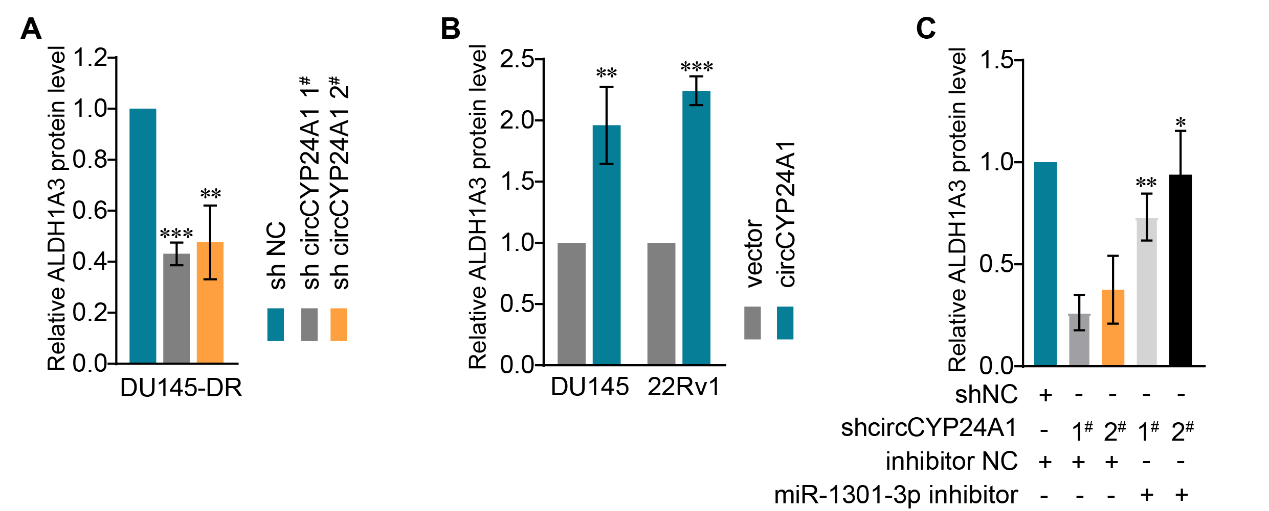
**

**Figure S3. The statistic diagram showed the quantification of the band intensity of ALDH1A3 protein level in in indicated cells. A.** ALDH1A3 protein level after knocking down circCYP24A1 expression in DU145-DR cells. **B.** ALDH1A3 protein level after upregulated circCYP24A1 expression in DU145 and 22RV1 cells. **C.** ALDH1A3 protein level after transfected with shcircCYP24A1 and miR1301-3p inhibitor in DU145-DR cells.
