# Supplementary material for: circCYP24A1 promotes Docetaxel resistance in prostate Cancer by Upregulating ALDH1A3
Source: Biomark Res. 2022 Jul 13;10:48. doi: 10.1186/s40364-022-00393-1 (PMC9277795; doi:10.1186/s40364-022-00393-1)
Supplement: Supplementary file 4 — Additional file 4: Figure S4. Representative images of IHC (ALDH1A3) staining of the xenograft tumors. [file 40364_2022_393_MOESM4_ESM.docx]

**Additional file 4: Figure S4**


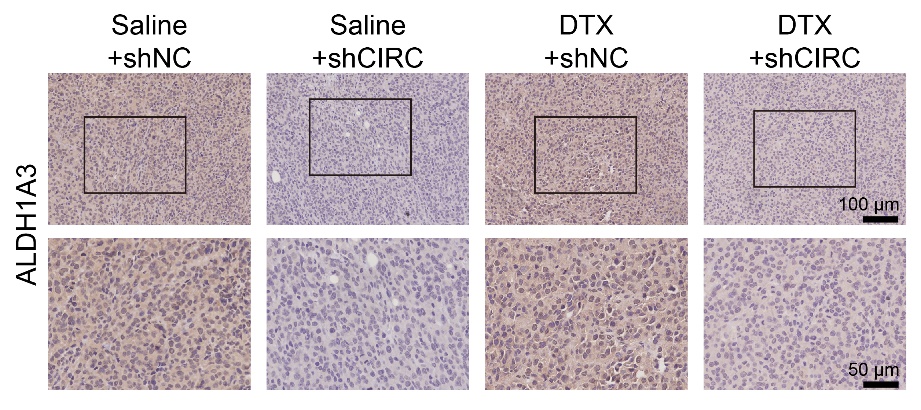


**Figure S4. Representative images of IHC (ALDH1A3) staining of the xenograft tumors.**
