# Supplementary material for: circCYP24A1 promotes Docetaxel resistance in prostate Cancer by Upregulating ALDH1A3
Source: Biomark Res. 2022 Jul 13;10:48. doi: 10.1186/s40364-022-00393-1 (PMC9277795; doi:10.1186/s40364-022-00393-1)
Supplement: Supplementary file 5 — Additional file 5: Figure S5. Two ALDH1A3 siRNAs were used to knock-down ALDH1A3 expression. Western blot was performed to validate si-ALDH1A3 efficiency. [file 40364_2022_393_MOESM5_ESM.docx]

**Additional file 5: Figure S5**


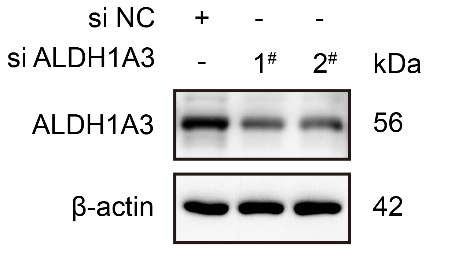


**Figure S5. Two ALDH1A3 siRNAs were used to knock-down ALDH1A3 expression.** Western blot was performed to validate si-ALDH1A3 efficiency.
