# Supplementary material for: circCYP24A1 promotes Docetaxel resistance in prostate Cancer by Upregulating ALDH1A3
Source: Biomark Res. 2022 Jul 13;10:48. doi: 10.1186/s40364-022-00393-1 (PMC9277795; doi:10.1186/s40364-022-00393-1)
Supplement: Supplementary file 6 — Additional file 6: Figure S6. Scatter plot shows the correlation among the level of circCYP24A1, miR-1301-3p and ALDH1A3. A. CircCYP24A1 with miR-1301-3p. (R = − 0.45, P < 0.001) B. circCYP24A1 with ALDH1A3. (R = 0.39, P < 0.001) C. MiR-1301-3p with ALDH1A3. (R = − 0.33, P = 0.0047). [file 40364_2022_393_MOESM6_ESM.docx]

**Additional file 6: Figure S6**

**
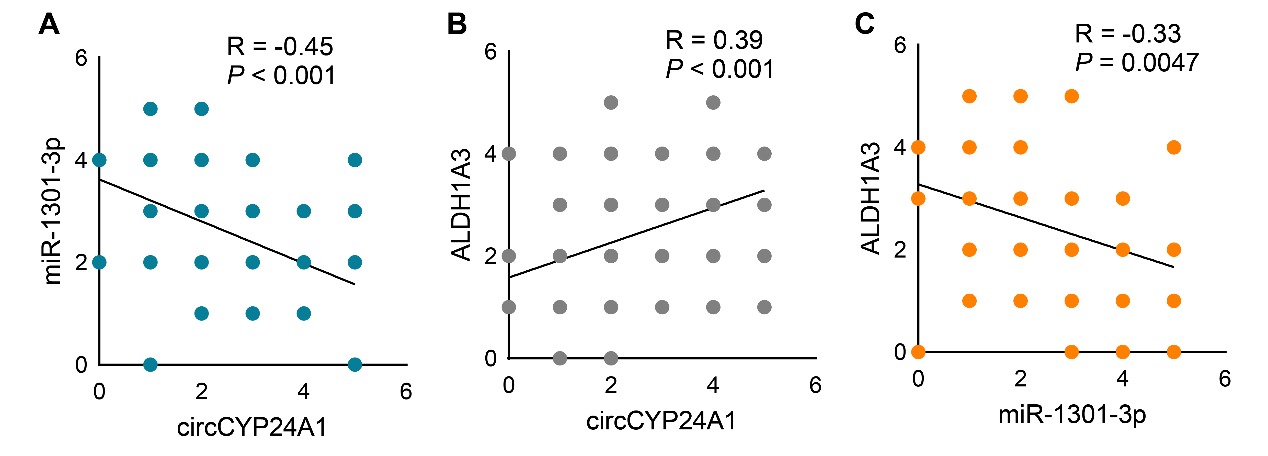
**

**Figure S6. Scatter plot shows the correlation among the level of circCYP24A1, miR-1301-3p and ALDH1A3. A.** CircCYP24A1 with miR-1301-3p. (R = -0.45, P<0.001) **B.** circCYP24A1 with ALDH1A3. (R = 0.39, P < 0.001) **C.** MiR-1301-3p with ALDH1A3. (R = -0.33, *P* = 0.0047)
