# Supplementary material for: circCYP24A1 promotes Docetaxel resistance in prostate Cancer by Upregulating ALDH1A3
Source: Biomark Res. 2022 Jul 13;10:48. doi: 10.1186/s40364-022-00393-1 (PMC9277795; doi:10.1186/s40364-022-00393-1)
Supplement: Supplementary file 7 — Additional file 7: Figure S7. ROC analysis to compare the diagnostic accuracy of the identified parameters. PSA = prostate specific antigen; AUC = area under the curve; 95% confidence intervals is labeled in parentheses. [file 40364_2022_393_MOESM7_ESM.docx]

**Additional file 7: Figure S7**

**
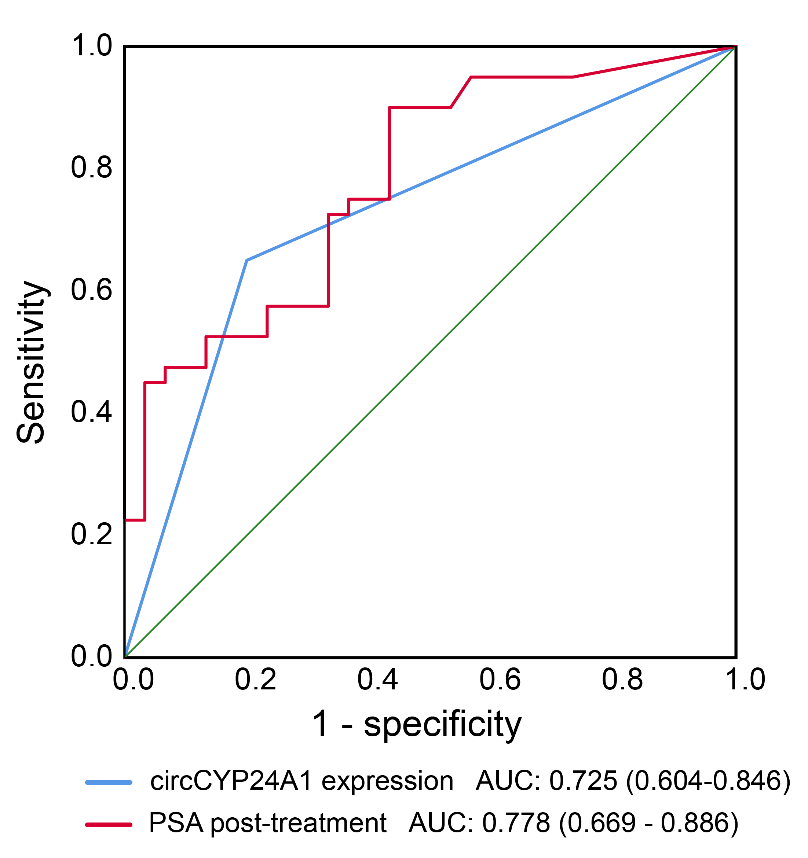
**

**Figure S7. ROC analysis to compare the diagnostic accuracy of the identified parameters.** PSA = prostate specific antigen; AUC=area under the curve; 95% confidence intervals is labeled in parentheses.
