# Supplementary material for: circCYP24A1 promotes Docetaxel resistance in prostate Cancer by Upregulating ALDH1A3
Source: Biomark Res. 2022 Jul 13;10:48. doi: 10.1186/s40364-022-00393-1 (PMC9277795; doi:10.1186/s40364-022-00393-1)
Supplement: Supplementary file 8 — Additional file 8: Table S1. Primers used for real-time PCR. [file 40364_2022_393_MOESM8_ESM.docx]

**Additional file 8: Table S1. Primers used for real-time PCR.**

| Target  gene | Primer sequence (5’-3’) | | Size  (bp) |
| --- | --- | --- | --- |
|  | Forward | Reverse |  |
| CircCYP24A1 | ATGCTACACTCAGGCACCCT | CCCCTTCCCTGAGGCGTATT | 81 |
| CYP24A1 | GGTGGCGAGACTCAGAACG | GTCGTGCTGTTTCTTGAGACC | 108 |
| ALDH1A3 | GAGCTGATCGAGAGTGGGAAG | GCTGTGAGTCCATAGTCGGT | 224 |
| ACTB | CATGTACGTTGCTATCCAGGC | CTCCTTAATGTCACGCACGAT | 250 |
| SRXN1 | CAGGGAGGTGACTACTTCTACTC | CAGGTACACCCTTAGGTCTGA | 126 |
| SKIL | GTTAAGCGAACCTGTACTTCTGT | GTAGGCGACATGCTTTCTTGG | 152 |
| MYBL1 | AGGCAAGCAGTGTAGAGAAAGA | CGATTTCCCAACCGCTTATGT | 117 |
| CHST3 | CATATCAAGGGTCTCAGACAAGC | GCGTTCTCAGCTAAGATCAGGG | 93 |
| NDRG3 | TCTTTCCCAACAGGGTATCAGT | GCTTAGGTGGGTAAGAACAGGA | 78 |
| CLU | CCAATCAGGGAAGTAAGTACGTC | CTTGCGCTCTTCGTTTGTTTT | 101 |
| PTPRJ | GGCACCCCTAGTCCAATTCC | TCCCATTAGATCCTTGTTCAGGT | 205 |
| FAM102B | CTTCGTCAATGGGGTCCTCTT | CACAGTTTGCTTGTACCACCT | 95 |
| MICAL3 | CCTGCAAGGGAACCCTCAAG | TGTGATAGAAGGAGCGGTAGTC | 84 |
| ARHGEF12 | ACACAGTCTACTATCACCGACA | TGCAATGCGCTCAACTTTCTG | 111 |
| UBL3 | AGTAATGTCCCGGCGGATATG | TCAGAAGCAGAATCGTTAGGAGA | 95 |
| TNS3 | ACCTCACTTACATCACGGAGC | GTAGGTTGTGCAGGTAGGACT | 81 |
| COL12A1 | AGCTGAGGCAGACATTGTGTT | CCTCCTTTGTACGGCAAGTTT | 237 |
| BNIP3L | ATGTCGTCCCACCTAGTCGAG | TGAGGATGGTACGTGTTCCAG | 189 |
| MXD4 | AACAGGTCTTCACACAACGAG | CTGCTCCTTGATGCTCAGTG | 195 |
| CircCYP24A1 ▷◁ | AGACCTGAACGTTGGCTTCA | TGAAGCTCTGCTAATCGGCG | 107 |
| CircCYP24A1 ◁▷ | ATGCTACACTCAGGCACCCT | CCCCTTCCCTGAGGCGTATT | 81 |
