# Supplementary material for: circCYP24A1 promotes Docetaxel resistance in prostate Cancer by Upregulating ALDH1A3
Source: Biomark Res. 2022 Jul 13;10:48. doi: 10.1186/s40364-022-00393-1 (PMC9277795; doi:10.1186/s40364-022-00393-1)
Supplement: Supplementary file 9 — Additional file 9: Table S2. Probes used for RNA pull-down. [file 40364_2022_393_MOESM9_ESM.docx]

**Additional file 9: Table S2.** **Probes used for RNA pull-down.**

| Target transcript | Probe sequence (5’-3’) |
| --- | --- |
| CircCYP24A1 probe | CUUCCCCUUCCUUUCAUUAGUUUCU |
| MiR-1301-3p | UUGCAGCUGCCUGGGAGUGACUUC |
| Negative Control | UUCAUCCAUUGCUUCUUCCCUCUUU |
