# Supplementary material for: circCYP24A1 promotes Docetaxel resistance in prostate Cancer by Upregulating ALDH1A3
Source: Biomark Res. 2022 Jul 13;10:48. doi: 10.1186/s40364-022-00393-1 (PMC9277795; doi:10.1186/s40364-022-00393-1)
Supplement: Supplementary file 10 — Additional file 10: Table S3. Primary antibodies used in this study. [file 40364_2022_393_MOESM10_ESM.docx]

**Additional file 10: Table S3. Primary antibodies used in this study.**

| Source | Primary antibodies | Catalog no. | Working dilution |
| --- | --- | --- | --- |
| ProteinTech | PI3K | 60225-1-Ig | WB: 1:5000 |
| ProteinTech | AKT | 60203-2-Ig | WB: 1:2000 |
| ProteinTech | mTOR | 66888-1-Ig | WB: 1:1000 |
| Cell Signaling Technology | p-mTOR | 5536 | WB: 1:1000 |
| Cell Signaling Technology | p-AKT | 4060 | WB: 1:2000 |
| ProteinTech | ALDH1A3 | 25167-1-AP | WB: 1:1000  IHC: 1:200 |
| Cell Signaling Technology | Cleaved caspase 3 | 9579 | IHC: 1:200 |
| Cell Signaling Technology | Ki67 | 9027 | IHC: 1:400 |
| Cell Signaling Technology | AGO2 | 2897 | RIP: 5μg |
