# Supplementary material for: circCYP24A1 promotes Docetaxel resistance in prostate Cancer by Upregulating ALDH1A3
Source: Biomark Res. 2022 Jul 13;10:48. doi: 10.1186/s40364-022-00393-1 (PMC9277795; doi:10.1186/s40364-022-00393-1)
Supplement: Supplementary file 12 — Additional file 12: Table S5. SiRNA used for silencing target genes. [file 40364_2022_393_MOESM12_ESM.docx]

**Additional file 12: Table S5.** **SiRNA used for silencing target genes.**

| Target transcript | Sequence (5’-3’) | |
| --- | --- | --- |
| CircCYP24A1 1# | AUAAUACGCCUCAGGGAAGTT |  |
| CircCYP24A1 2# | GAUAAUACGCCUCAGGGAATT |  |
| ALDH1A3 1# | GGGCCUCAGAUUGAUCAAATT |  |
| ALDH1A3 2# | GCAGAGAACUAGGUGAAUATT |  |
