# Supplementary material for: circCYP24A1 promotes Docetaxel resistance in prostate Cancer by Upregulating ALDH1A3
Source: Biomark Res. 2022 Jul 13;10:48. doi: 10.1186/s40364-022-00393-1 (PMC9277795; doi:10.1186/s40364-022-00393-1)
Supplement: Supplementary file 13 — Additional file 13: Table S6. Baseline characteristics of the included 70 high-risk PCa patients who received neoadjuvant therapy with DTX. [file 40364_2022_393_MOESM13_ESM.docx]

| **Additional file 13: Table S6. Baseline characteristics of the included 70 high-risk PCa patients who received neoadjuvant therapy with DTX.** | |
| --- | --- |
|  |  |
| Characteristics | Value |
| Age (years) | 69 (63-74) |
| PSA pre-treatment (ng/ml) | 47.0 (20.5-127.5) |
| Prostate volume(ml) | 16.9 (14.6-21.3) |
| PSA post-treatment (ng/ml) | 0.10 (0.02-0.46) |
| Tumor diameter on MRI | 2.4 (1.3-3.2) |
| PI-RADS |  |
| 3 | 5 (7.1) |
| 4 | 18 (25.7) |
| 5 | 47 (67.1) |
| Clinical T stage |  |
| T1/T2 | 0 (0) |
| T3a | 26 (37.1) |
| T3b | 36 (51.4) |
| T4 | 8 (11.4) |
| ISUP grade at biopsy |  |
| 1 | 7 (10.0) |
| 2 | 11 (15.7) |
| 3 | 8 (11.4) |
| 4 | 30 (42.9) |
| 5 | 14 (20.0) |
| Pathological T stage |  |
| T1/T2 | 26 (37.1) |
| T3a | 15 (21.4) |
| T3b | 29 (41.4) |
| T4 | 0 (0) |
| Continuous variables are presented as median (interquartile range, IQR), while categorical variables are presented as patients (%) | |
| MRI = magnetic resonance imaging; PSA = prostate-specific antigen; ISUP = International Society of Urological Pathology; PI-RADS = Prostate Imaging Reporting and Date System | |
